# Low-dose decitabine enhances the efficacy of viral cancer vaccines for immunotherapy

**DOI:** 10.1016/j.omton.2024.200766

**Published:** 2024-01-26

**Authors:** Salvatore Russo, Sara Feola, Michaela Feodoroff, Jacopo Chiaro, Gabriella Antignani, Manlio Fusciello, Federica D’Alessio, Firas Hamdan, Teijo Pellinen, Riikka Mölsä, Lorella Tripodi, Lucio Pastore, Mikaela Grönholm, Vincenzo Cerullo

**Affiliations:** 1 Drug Research Program (DRP), ImmunoViroTherapy Lab (IVT), Division of Pharmaceutical Biosciences, Faculty of Pharmacy, University of Helsinki, Viikinkaari 5E, 00790 Helsinki, Finland; 2 Helsinki Institute of Life Science (HiLIFE), University of Helsinki, Fabianinkatu 33, 00710 Helsinki, Finland; 3 Translational Immunology Program (TRIMM), Faculty of Medicine Helsinki University, University of Helsinki, Haartmaninkatu 8, 00290 Helsinki, Finland; 4 Digital Precision Cancer Medicine Flagship (iCAN), University of Helsinki, 00014 Helsinki, Finland; 5 Institute for Molecular Medicine Finland (FIMM), HiLIFE, University of Helsinki, Helsinki, Finland; 6 Department of Molecular Medicine and Medical Biotechnology and CEINGE, Naples University, 24 Federico II, 80131 Naples, Italy; 7 CEINGE-Biotecnologie Avanzate Franco Salvatore s.c.a.r.l, 80131 Naples, Italy

**Keywords:** MT: Regular Issue, cancer vaccines, immunotherapy, epigenetic therapy, oncolytic vaccines, oncolytic viruses

## Abstract

Cancer immunotherapy requires a specific antitumor CD8^+^ T cell-driven immune response; however, upon genetic and epigenetic alterations of the antigen processing and presenting components, cancer cells escape the CD8^+^ T cell recognition. As a result, poorly immunogenic tumors are refractory to conventional immunotherapy. In this context, the use of viral cancer vaccines in combination with hypomethylating agents represents a promising strategy to prevent cancer from escaping immune system recognition. In this study, we evaluated the sensitivity of melanoma (B16-expressing ovalbumin) and metastatic triple-negative breast cancer (4T1) cell lines to FDA-approved low-dose decitabine in combination with PeptiCRAd, an adenoviral anticancer vaccine. The two models showed different sensitivity to decitabine *in vitro* and *in vivo* when combined with PeptiCRAd. In particular, mice bearing syngeneic 4T1 cancer showed higher tumor growth control when receiving the combinatorial treatment compared to single controls in association with a higher expression of MHC class I on cancer cells and reduction in Tregs within the tumor microenvironment. Furthermore, remodeling of the CD8^+^ T cell infiltration and cytotoxic activity toward cancer cells confirmed the effect of decitabine in enhancing anticancer vaccines in immunotherapy regimens.

## Graphical abstract

**Figure:**
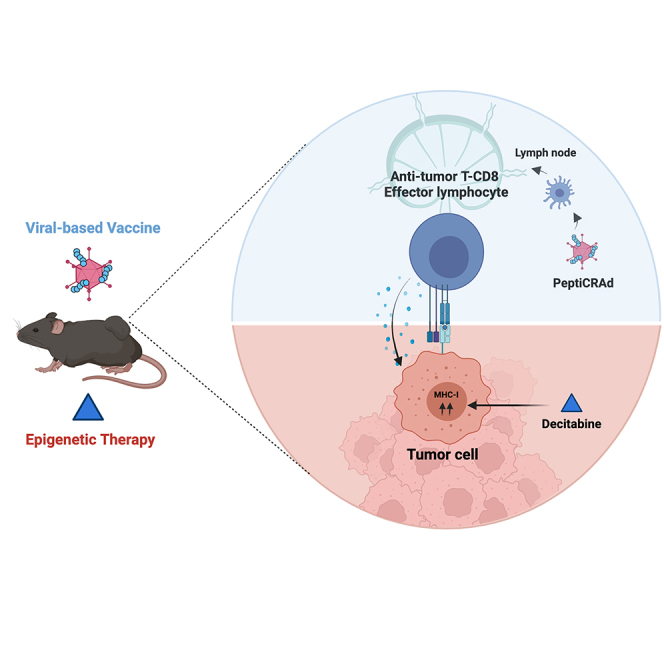

## Teaser

In this study, Cerullo and colleagues evaluated the effect of FDA-approved decitabine (DAC) in enhancing the activity of the viral-based cancer vaccine PeptiCRAd. Synergistically, PeptiCRAd and DAC generated an enhanced specific antitumor immune response in a murine model of triple-negative breast cancer.

## Introduction

Oncolytic viruses (OVs) are genetically modified or naturally occurring viruses able to kill cancer cells selectively. Besides directly killing tumor cells, OVs act as immune adjuvants to promote intratumoral inflammation by stimulating both innate and adaptive immunity. Hence, OV-based adjuvants represent an attractive and promising platform for cancer vaccine development to be included in immunotherapy-based regimens (e.g., in combination with immune checkpoint inhibitors [ICIs]).^1^ However, most patients show limited response to such regimens. Among several reasons for this phenomenon, cancer develops genetic and epigenetic alterations that impair major histocompatibility complex class I (MHC class I) expression and the antigen processing machinery (APM), hampering the CD8^+^ T cells/T cell receptor–cancer interaction^2^^,^^3^; however, cancer cells modulate the immune system both at the systemic and the local level, by driving T cells’ and other immune effectors’ exhaustion and limiting their activation. Subsequently, immunotherapy-based approaches require novel strategies to tackle the molecular events responsible for poor clinical outcomes.

Interestingly, hypomethylating agents (HMAs) have shown great potential in remodeling cancer gene expression and increasing cancer immunogenicity.^4^^,^^5^^,^^6^ For example, low-dose US Food and Drug Administration (FDA)-approved HMAs azacytidine and decitabine (DAC) positively modulate the immune profile and outcome in cancer patients and—noteworthy—correlate with a better response when combined with ICIs.^7^^,^^8^^,^^9^^,^^10^ Furthermore, low-dose HMAs have been associated with enhanced MHC class I and APM expression,^5^ as well as induction of tumor antigens (TAs) and retroviral-derived antigens.^11^ These elements account for increased tumor immunogenicity and potentially sensitize hard-to-treat cancers to immunotherapy. Interestingly, HMAs are able to remodel the intratumoral immune infiltration. In particular, besides enhancing the CD8 T cell-mediated response, HMAs are involved in the depletion of immune suppressor cells, such as regulatory T cells (Tregs) and myeloid-derived suppressive cells (MDSCs), and are currently evaluated in several clinical trials as immune adjuvants for sensitizing cancer patients to checkpoint inhibitor immunotherapy.^12^^,^^13^^,^^14^ For these reasons, we examined the potential synergism between HMAs and immunovirotherapy to improve anticancer vaccine efficacy.

In this study, we examined the effect of the FDA-approved DAC on MHC class I and programmed death-ligand 1 (PD-L1) expression levels in cancer cell lines. Then, to evaluate epigenetic therapy in enhancing an antiumor-specific immune response, we tested whether low-dose DAC could improve the anticancer vaccine platform PeptiCRAd (peptides-coated conditionally replicating adenovirus) in murine models of melanoma and triple-negative breast cancer (TNBC). Briefly, PeptiCRAd takes advantage of the electrostatic association between tumor peptides and a human oncolytic adenovirus-5/3 Δ24 (OAd). Despite the lack of the oncolytic effect of human OAds in murine cancer cells, PeptiCRAd acts as an anticancer vaccine by combining (1) the adjuvant effect provided by the virus and (2) the antigen/peptide coated on its surface. In these settings, PeptiCRAd induces specific antitumor immunity^15^^,^^16^^,^^17^ in mice independently from viral replication and is currently evaluated in a Phase I clinical trial for the treatment of melanoma, triple-negative breast cancer, and non-small cell lung cancer.^18^ This study was registered at Clinicaltrials.gov: NCT 05492682.

## Results

### Low-dose DAC-mediated MHC class I and PD-L1 expression via epigenetic remodeling is dependent on the tumor type

To investigate the effect of epigenetic remodeling as a priming agent to potentiate cancer immunotherapeutic approaches, we first evaluated the effect of DAC on MHC class I and PD-L1 expression in low-MHC class I-expressing melanoma and high-MHC class I-expressing TNBCs cell lines.^19^ As models, B16-OVA (murine model of melanoma-expressing ovalbumin), A-375 (human model of melanoma), 4T1 (murine model of TNBC) and MDA-MB436 (human model of TNBC) cancer cell lines were evaluated.

**Figure 1:**
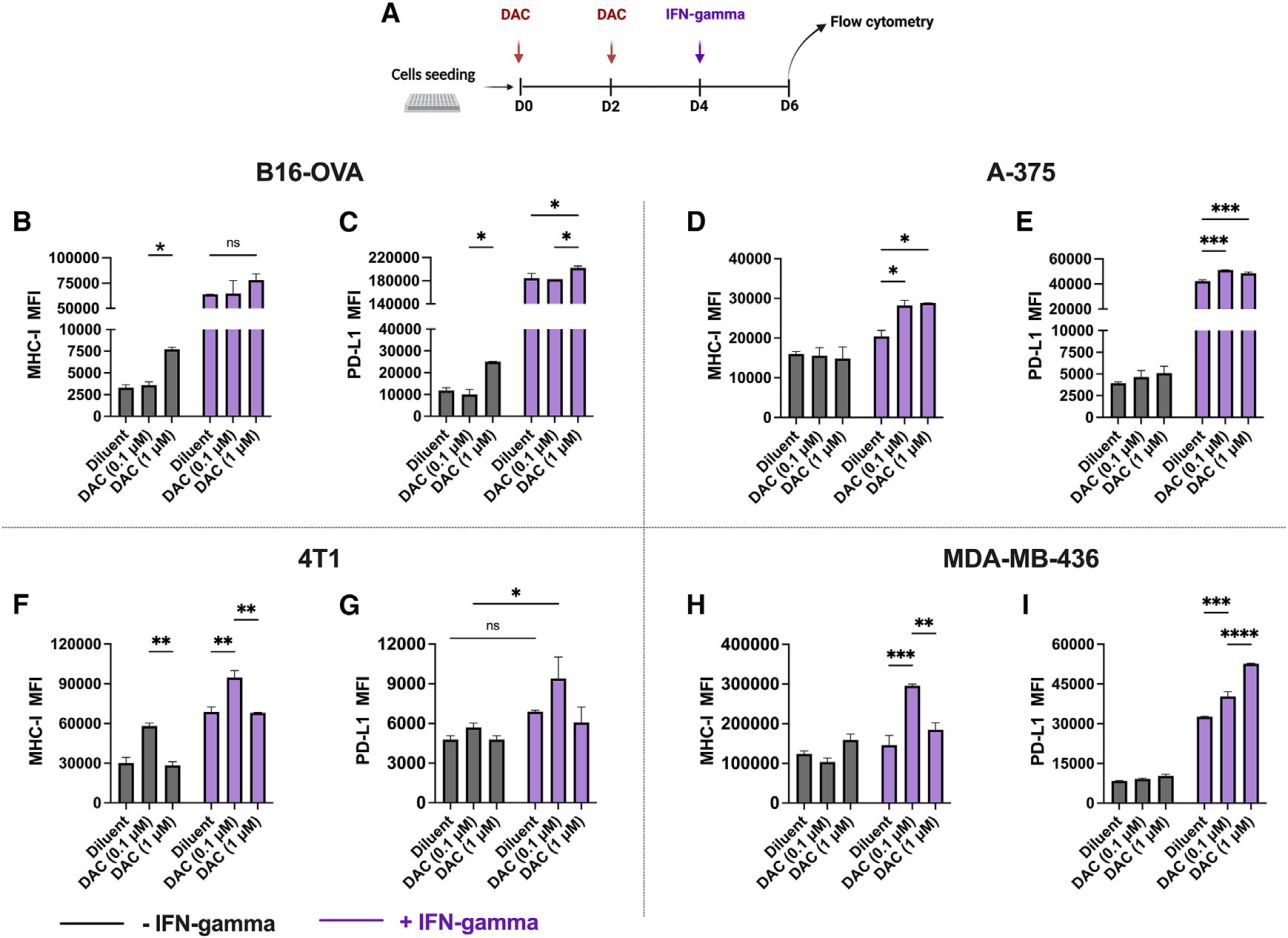
Expression level of MHC-I and PD-L1 in melanoma (human, A-375; murine B16-OVA) and TNBC (human, MDA-MB-436; murine, 4T1) cell lines (A) The cells were stimulated with 0.1 μM (low dose) or 1 μM (high dose) DAC for 4 days, followed by the addition of IFN-γ as shown in schematic (A). (B–I) MFI of MHC class I (B–E) and PD-L1 (F–I) were then analyzed by flow cytometry using anti-mouse or anti-human fluorophore-conjugated antibodies. N = 3 in (B)–(I). Levels of significance were set at ∗p < 0.05, ∗∗p < 0.01, ∗∗∗p < 0.001, and ∗∗∗∗p < 0.0001 (2-way ANOVA with Tukey’s multiple comparisons correction to compare individual groups). Graphs are shown as mean ± SD.

We treated the cells with 0.1 μM (low dose) or 1 μM DAC (high dose) or with diluent (PBS) at days 0 and 2, followed by interferon-γ (IFN-γ) stimulation at day 4 (Figure 1A); at the end of the treatment, cells were stained, and the mean fluorescence intensity (MFI) of MHC class I and PD-L1 was evaluated by flow cytometry. In the absence of IFN-γ, both high-dose and low-dose DAC significantly induced MHC class I expression in B16-OVA (Figure 1B) and 4T1 (Figure 1C), respectively. Furthermore, DAC-mediated priming resulted in higher MHC class I levels when followed by IFN-γ at both doses in A-375 (Figure 1D) and only at low doses in human MDA-MB-436 (Figure 1E). Nevertheless, PD-L1 followed the MHC class I induction pattern in all of the cell lines (Figures 1F–1H), except for MDA-MB-436, where levels increased dose dependently in the presence of IFN-γ (Figure 1I). It is worth noting that IFN-γ boosted the percentage of cells expressing MHC class I and PD-L1 expression levels in all of the cell lines except for 4T1 cells (Figures S1A‒S1D),^20^ whereas low-dose DAC treatment effectively reduced whole 5-methylcytosine in B16.OVA and 4T1 cells (Figures S1E and S1F). These data confirm the ability of DAC to promote MHC class I and PD-L1 expression and further enhance it in the presence of IFN-γ on the surface of the cancer cell’ in a tumor-specific manner.

### Low-dose DAC does not increase human OAd cytotoxicity in murine and human cancer cells

**Figure 2:**
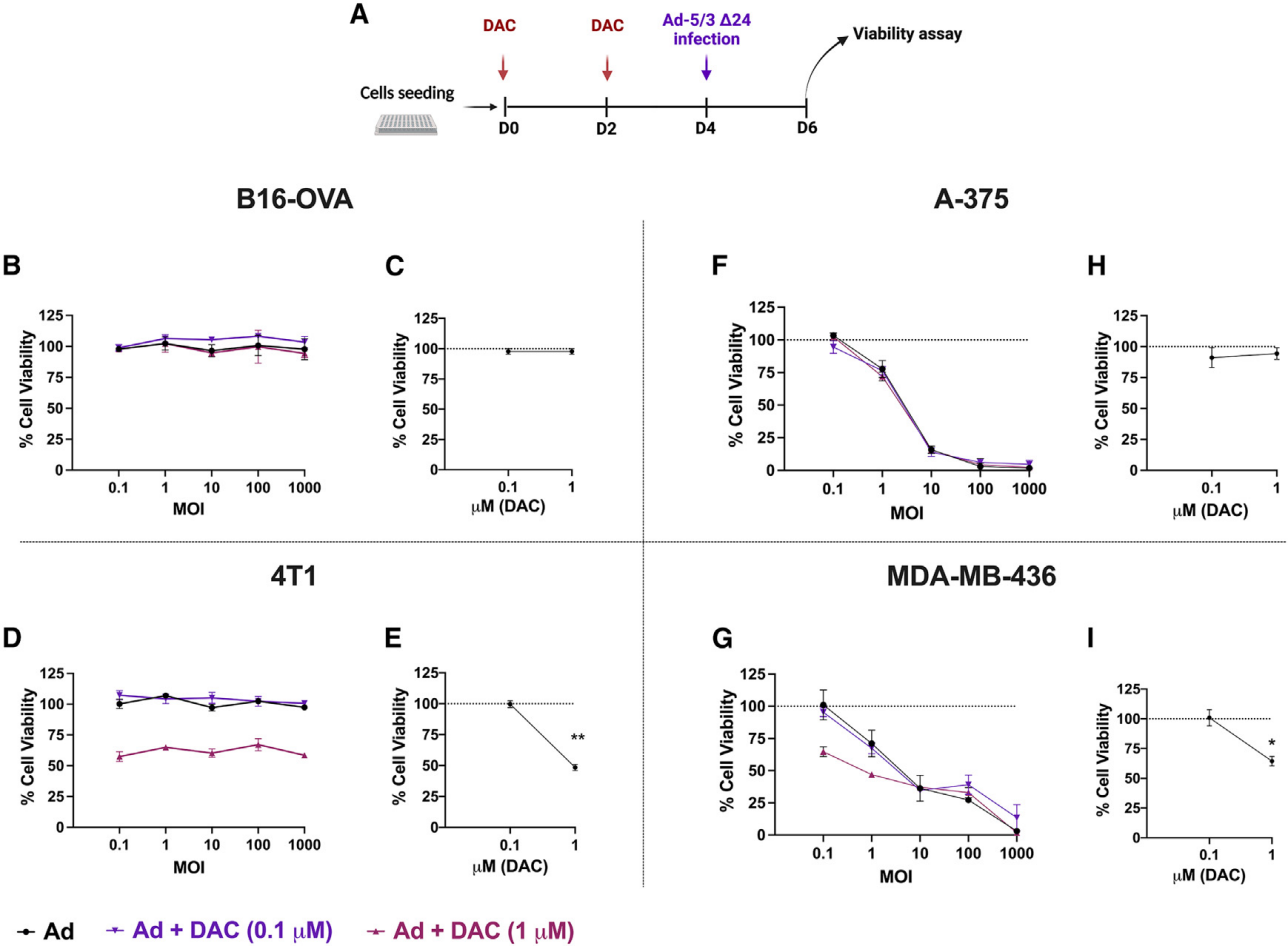
Cell viability in in melanoma (human, A-375; murine B16-OVA) and TNBC (human, MDA-MB-436; murine, 4T1) cell lines (A) The cells were stimulated with 0.1 μM (low dose) or 1 μM (high dose) of DAC for 4 days and followed by the infection with Ad-5/3 Δ24 (Ad). (B–I) Cell viability assay was evaluated using MTS assay. The cells were infected with Ad5/3Δ24 (Ad) (black), Ad + DAC 0.1 μM (purple), or Ad + DAC 1 μM (pink) at different MOIs, as indicated in (B), (D), (F), and (G). (C), (E), (H), and (I) represent cell viability after different doses of DAC (0.1 and 1 μM). N = 4. Levels of significance were set at ∗p < 0.05, ∗∗p < 0.01, ∗∗∗p < 0.001, and ∗∗∗∗p < 0.0001 (2-way ANOVA with Tukey’s multiple comparisons correction to compare individual groups). Graphs are shown as mean ± SD.

To investigate the potential synergism between epigenetic therapy and the viral cancer vaccines, we evaluated whether the simultaneous presence of human OAd and epigenetic therapy could affect the viability of cancer cells. We chose B16-OVA and 4T1 cells as murine models and A-375 and MDA-MB-436 as human models for testing viral replication. Unlike human cells and cell lines, murine cells show infection resistance and do not allow the formation of competent virions after infection with human-derived OAd. Thus, cells were primed with different doses of DAC (0.1 μM, low dose; 1 μM, high dose) and then infected them with different MOIs using human OAd (Figure 2A). As expected, OAd did not show any toxic effect in murine cells. B16-OVA showed 100% viability when treated with OAd alone or in combination with DAC (Figures 2B and 2C). Similarly, 4T1 cells were not affected by OAd alone or 0.1 μM DAC; however, 1 μM DAC reduced cell viability (48%) either in the presence of different OAd MOIs (Figure 2D) or alone (Figure 2E). Conversely, A-375 cells allowed Ad-5/3 Δ24 replication with a subsequent MOI-dependent effect on cell viability (Figure 2F); however, as shown in B16-OVA cells, the human melanoma was insensitive to DAC treatments (Figure 2H). Reduced cell viability was reported only in the presence of 1 μM DAC combined with OAd at 0.1 (64%) and 1 MOI (47% viability) in MDA-MB-436 cells (Figure 2G) or alone (65%) (Figure 2I). At higher MOIs, no synergistic effect was observed at high doses, whereas 0.1 μM DAC did not affect the viability state either in combination with OAd or as a single agent (Figure 2I).

### Rational design of low-dose DAC and viral cancer vaccines increases tumor growth control in TNBC

OVs are well-known immune stimulators and recruit several immune players within the tumor microenvironment (TME).^21^ Despite the lack of replication in murine cancer cells, the adjuvant effect provided by OAd in the PeptiCRAd complex can trigger anticancer T cell immune responses toward a specific TA.^16^^,^^17^^,^^18^^,^^22^ Simultaneously, demethylating agents enhance MHC class I expression levels and immunogenicity of cancer cells, potentially supporting the cytotoxic CD8^+^ T cell activation within the TME. Therefore, we hypothesized that the combination of these two strategies would result in better antitumor efficacy than the single agents in mice *in vivo*.

**Figure 3:**
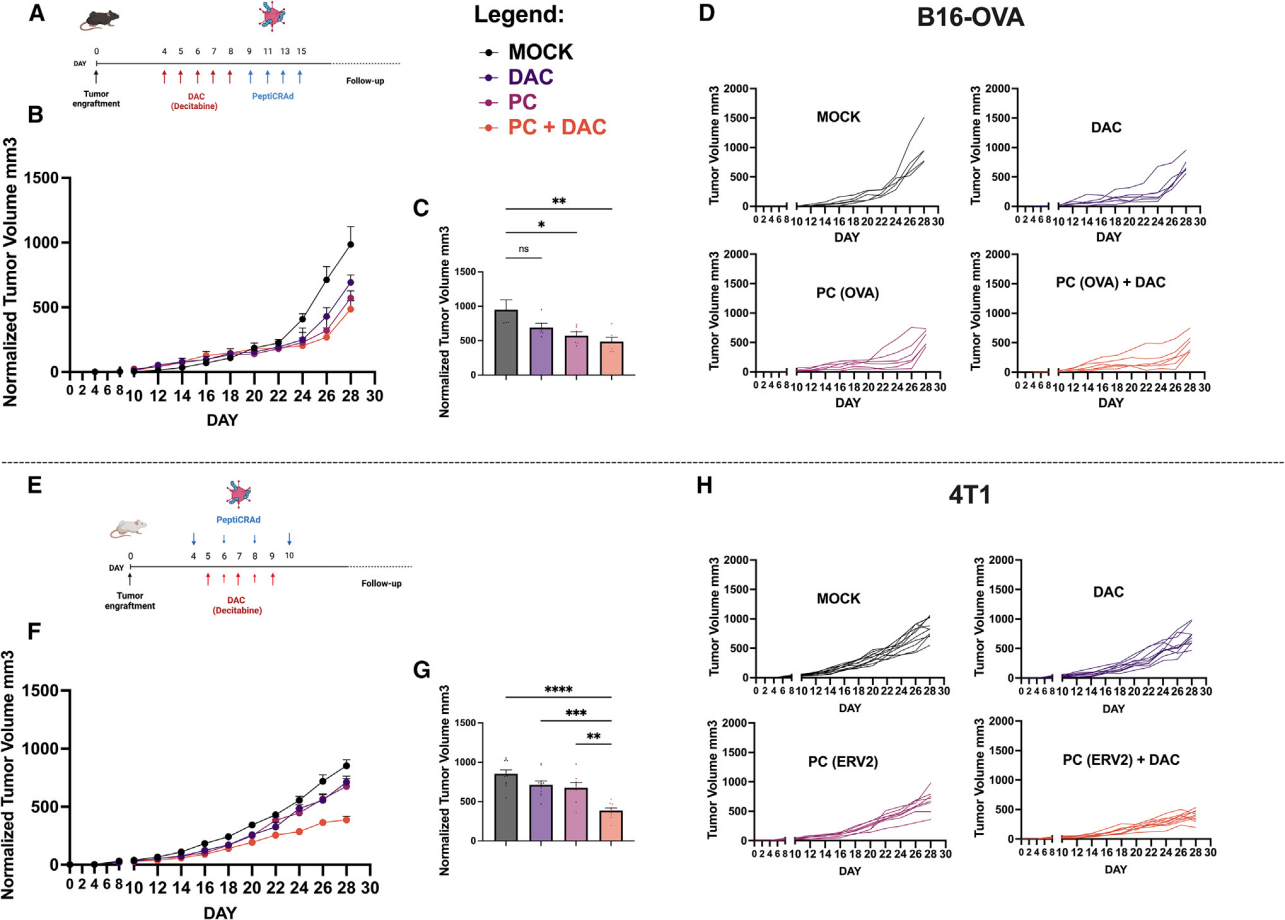
Tumor growth in syngeneic mouse model B16-OVA and 4T1 Treatment schematics are depicted in (A) and (E) for B16-OVA and 4T1, respectively. Normalized tumor growth curves for each model are shown in (B) and (F); the number of mice in the B16-OVA model was 5 in the MOCK group and 7 in the others; the number of mice in the 4T1 model was 8–10. Tumor volumes were normalized on day 4 measurements. (C) and (G) show the volumes that the tumors from each treatment reached at day 28 in B16-OVA and 4T1, respectively. The graphs in (D) and (H) show single tumor growth curves for each treatment group. Error bars represent SEMs. Levels of significance were set at ∗p < 0.05, ∗∗p < 0.01, ∗∗∗p < 0.001, and ∗∗∗∗p < 0.0001 (2-way ANOVA with Tukey’s multiple comparisons correction to compare individual groups).

To this end, we subcutaneously (s.c.) injected immunocompetent mice with B16-OVA, a poorly immunogenic^23^ murine model of melanoma (day 0). Then, 4 days after tumor engraftment, mice received daily intraperitoneally (i.p.) injections of a low-dose DAC (0.5 mg/kg) for 5 sequential days followed by 4 intratumoral PeptiCRAd administrations (starting from day 9); in particular, mice received PeptiCRAd coated with SIINFEKL peptide (PC(OVA)) from days 9 to 15 every 2 days (Figure 3A). At the end of the experiment, tumor size averages were calculated. As a result, only PeptiCRAd and PeptiCRAd+ DAC treatments showed tumor growth control. However, although associated with reduced tumor size, the combination of PeptiCRAd and DAC was not significantly different from the single treatments (Figures 3B‒3D). To test whether a relevant effect on tumor growth could be achieved in a highly aggressive and metastatic model,^23^ we s.c. implanted 4T1 cells in syngeneic Balb/c mice. In this model, mice received epigenetic therapy and PeptiCRAd in the same early therapeutic window to obtain more time points for tumor growth measurement after the last treatment. Therefore, PeptiCRAd was administrated s.c. in the area surrounding the tumors every 2 days from days 4 to 10 posttumor cell injection engraftment. In addition, DAC was given i.p. from days 5 to 10 daily (Figure 3E). For the PeptiCRAd design, we tested the ERV2 peptide that had been previously identified and characterized by direct immunopeptidomic analysis in the 4T1 cell line.^16^ Groups treated with DAC or PC(ERV2) as single agents showed a limited and not significant response; however, when combined with DAC, PeptiCRAd showed a consistent control of the tumor growth (Figures 3F‒3H). Furthermore, we retested the combination-based approach in the 4T1 model, obtaining similar results in terms of tumor growth control by using two additional peptides expressed by 4T1 cells: AH1 (contains the heteroclitic peptide AH1-5 that derives from the gp70 epitope^24^) and ERV1^16^ (Figure S3).

### DAC increases the expression of MHC class I and PD-L1 on TBNC cells *in vivo*

**Figure 4:**
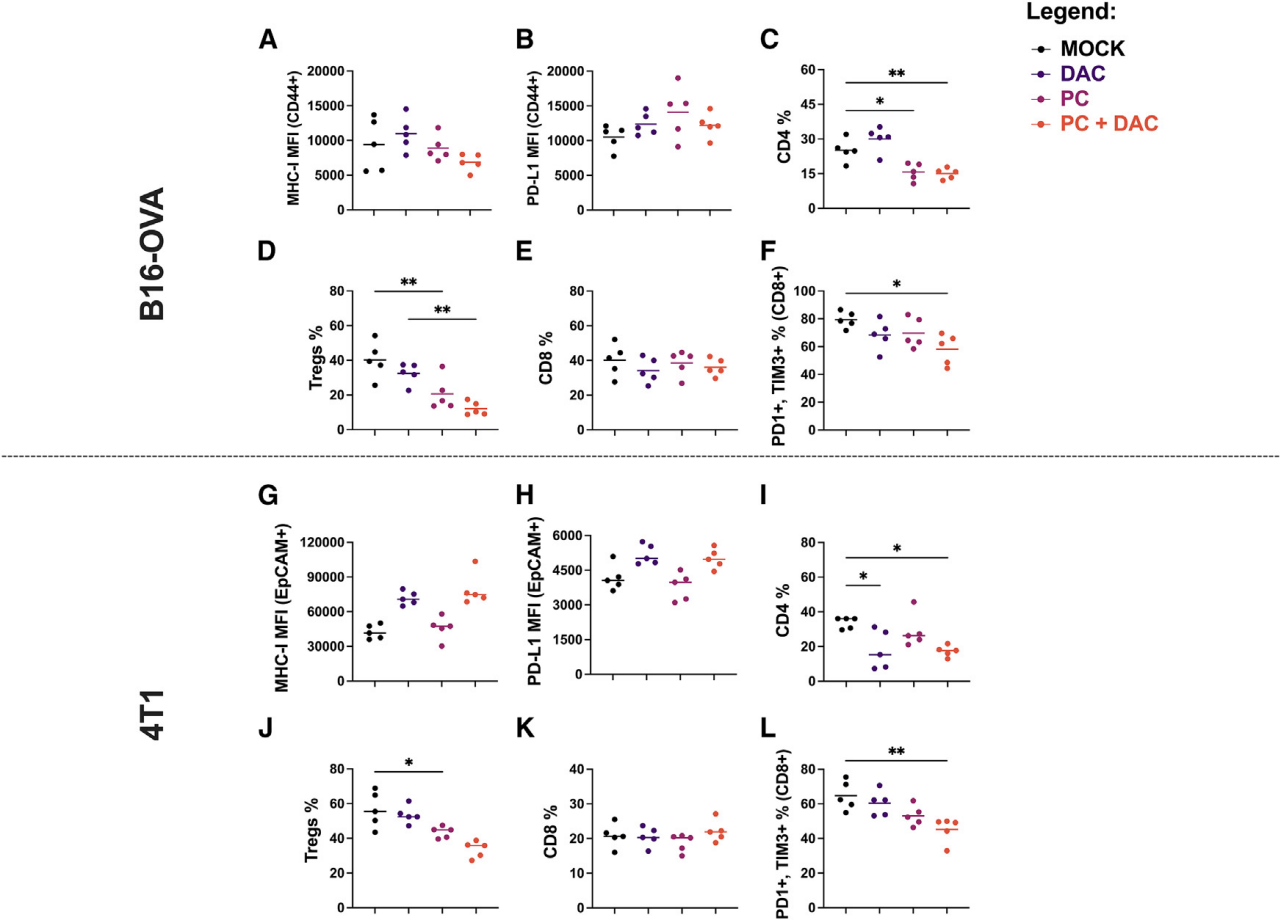
Intratumoral expression of MHC class I and PD-L1 and lymphocyte infiltration Intratumoral expression MHC-I (A and G), PDL-1 (B and H), CD4 (C and I), Tregs (D and J), CD8 (E and K), and exhaustion markers (PD-1, TIM-3). (F and L) Exhaustion markers were evaluated within the TME in B16-OVA and 4T1. N = 5; each dot represents a single mouse. Horizontal bar represents the mean. Levels of significance were set at ∗p < 0.05, ∗∗p < 0.01, ∗∗∗p < 0.001, and ∗∗∗∗p < 0.0001 (2-way ANOVA with Tukey’s multiple comparisons correction to compare individual groups).

To better elucidate the mechanisms underpinning the enhanced activity observed in the 4T1 model, we decided to characterize the expression of MHC class I and PD-L1 on harvested tumors, and the lymphocyte infiltration within the TME in both experiments by flow cytometry. In the B16-OVA model, no statistically significant difference was observed among the treatments when we investigated the MHC class I (Figure 4A) or PD-L1 (Figure 4B) expression on cancer cells (CD44^+^, CD3^−^). Regarding the tumor infiltrating lymphocytes (TILs) compartment (Figure S2A), PeptiCRAd and PeptiCRAd+ DAC, despite a lower CD4^+^ T cell infiltration (Figure 4C), showed a reduced percentage of intratumoral Tregs (CD25^+^, FOXP3^+^) (Figure 4D). It is interesting to note that B16-OVA tumors showed no difference in CD8^+^ T cell infiltration (Figure 4E), whereas coexpression of PD-1 and T cell immunoglobulin and mucin domain-containing protein 3 (TIM-3) exhaustion-associated molecules on CD8^+^ T cells were reduced in the combination-based group (Figure 4F).

In the 4T1 model, however, mice receiving DAC either alone or in combination with PeptiCRAd significantly enhanced MHC class I (Figure 4G) and PD-L1 (Figure 4H) expression on cancer cells (EP-CAM^+^, CD3^−^), in contrast with the previous B16-OVA animal experiment. On the TILs side (Figure S2B), although 4T1 tumors treated with DAC alone or in combination showed a significant reduction in CD4^+^ T cells (Figure 4I), only PeptiCRAd-based treatments reduced the Tregs cell (CD25^+^, FOXP3^+^) compartments (Figure 4J), as observed in B16-OVA TILs. We then noticed that in the absence of significant change in CD8^+^ T infiltration (Figure 4K), PeptiCRAd in combination with DAC showed the lowest coexpression of PD-1 and TIM-3 on CD8^+^ T cells (Figure 4L). In addition, we analyzed mice spleens for systemic immune profiling. Here, no significant impact on T lymphocyte populations among the treatment groups was observed in B16-OVA (Figure S4A), whereas the 4T1 mice receiving the combination showed a significant increase in CD8^+^ and CD4^+^ T cells but not Tregs (Figure S4B). Furthermore, we analyzed with flow cytometry the MDSCs in the TME at the endpoint (Figure S4C). Granulocytic MDSCs (G-MDSCs) and monocytic MDSCs (M-MDSCs) are known to populate the TME of 4T1 tumors and mediate immunosuppression, reducing the sensitivity of this tumor to cancer immunotherapy. In our groups, mice receiving DAC (alone or in combination with PeptiCRAd) showed a decrease in the percentage of G-MDSCs (Ly6C^+^, Ly6G^+^) and M-MDSCs (Ly6C^+^, Lys6G^−^) as also previously shown.^12^

### PeptiCRAd+ DAC remodels the CD8^+^ T cell infiltration pattern within the 4T1 TME

**Figure 5:**
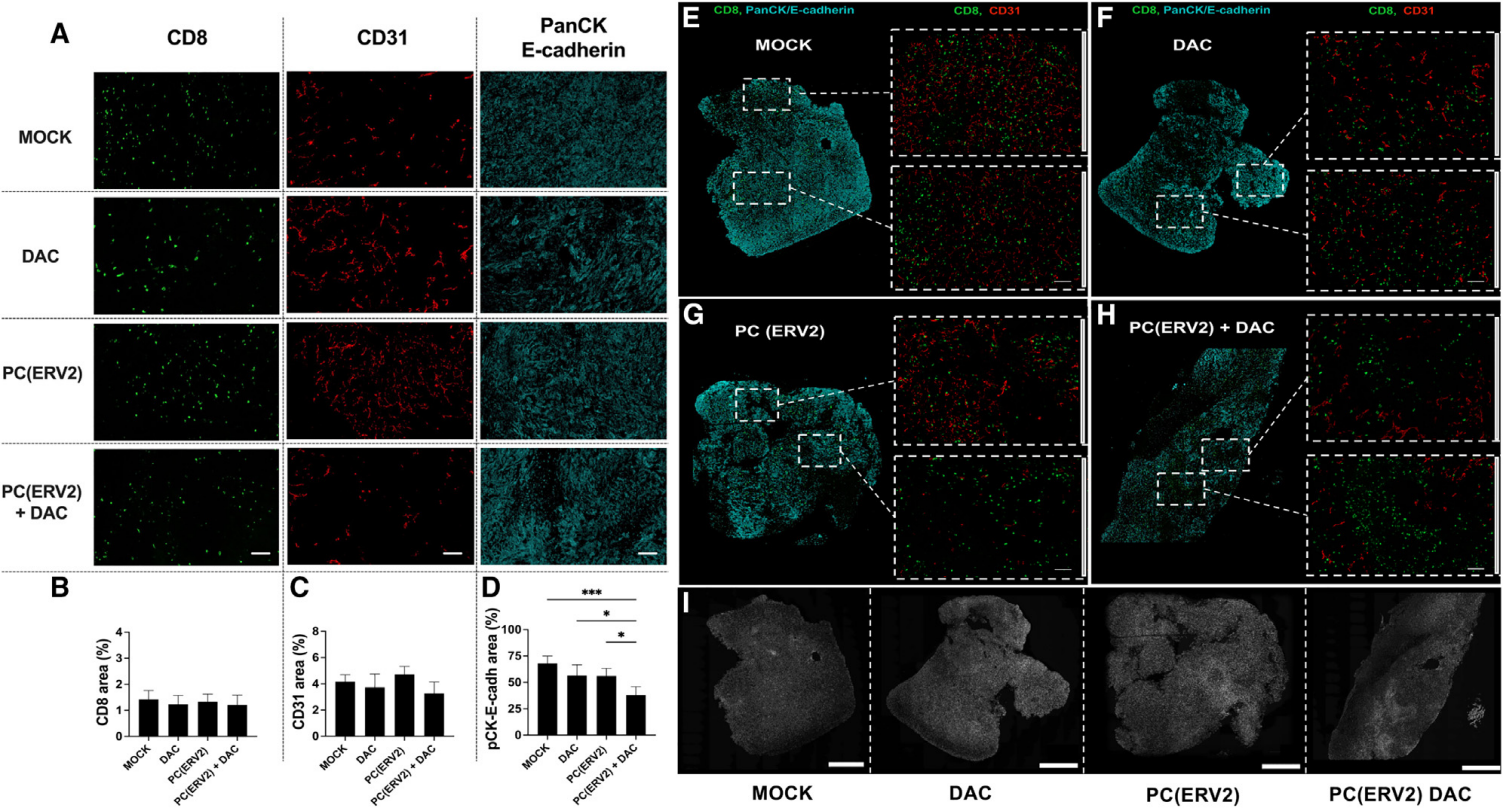
Multiplex IHC of 4T1 tumors Representative images from the 4T1 murine tumors were antibody stained with lymphocyte (CD8), endothelial (CD31), and tumor (pan-CK and E-cadherin) markers (A). In addition, all of the histological sections were counterstained with H&E. Graphs are shown as mean ± SD. N = 10 pictures were analyzed from 3 different tumors. The histograms (B–D) show the mean value for each signal area percentage. Levels of significance were set at ∗p < 0.05, ∗∗p < 0.01, ∗∗∗p < 0.001, and ∗∗∗∗p < 0.0001 (1-way ANOVA with Tukey’s multiple comparisons correction to compare individual groups). (E–H) show a whole-tumor section stained with CD8 and pan-CK/E-cadherin. Representative regions for CD8 and CD31 staining are displayed for each tumor section from each treatment group (bar, 100 μm). (I) represents the DAPI staining of the tumor sections (bar, 500 μm).

Although both epigenetic agents and OVs have been previously associated with higher CD8 infiltration,^22^^,^^25^ surprisingly, the treatments did not induce this effect in the 4T1 tumors. Nevertheless, the combination group showed CD8 levels similar to those of untreated mice, despite being associated with a reduction in the tumor volume. Therefore, we reexamined the CD8 intratumoral presence by immunohistochemistry (IHC) to assess whether our treatments affected the tumor architecture and to elucidate the role of CD8 in the 4T1 tumor reduction. To this aim, we used formalin-fixed paraffin-embedded (FFPE) murine 4T1 tumors and simultaneously evaluated CD8 (cytotoxic T cell marker), CD31 (blood vessels marker, for tracking the CD8 infiltration pattern), and pan-cytokeratin (pan-CK)/E-cadherin (tumor markers)^26^ (N = 12 images from 3 mice per group were analyzed). The CD8 levels were similar among the different treatments (Figure 5A), confirming the fluorescence-activated cell sorting results; 4T1 is indeed known to be infiltrated by lymphocytes despite high levels of immune suppression.^23^ Similarly, CD31 expression showed no difference as well (Figure 5B). However, as expected, PC(ERV2)+ DAC resulted in the lowest density of pan-CK/E-cadherin (Figure 5C). However, when looking at the tumor architecture, we observed the presence of nonnecrotic high- and low-density pan-CK/E-cadherin areas in the whole-tumor sections (Figures 5E–5I). For example, in both high-density (Figure S5) and low-density (Figure S6) pan-CK/E-cadherin areas from untreated (MOCK), DAC, and PC(ERV2), CD8 cells correlated in terms of quantity and localization with the tumor blood vessels. Besides this pattern, the PC(ERV2) + DAC tumors were characterized by CD8 clusters that were localized in low-density pan-CK/E-cadherin areas and not associated with the presence of blood vessels (CD31) (Figures 5H and S6). The absence of such clusters and the presence of a blood vessel-dependent CD8 localization in MOCK, DAC, and PC(ERV2) tumors suggests a reorganization of the intratumoral CD8^+^ T cell infiltration pattern in addition to tumor stroma reduction upon PC(ERV2) + DAC treatment.

### PeptiCRAd, in combination with DAC, resulted in higher specific antitumor T cell activation

**Figure 6:**
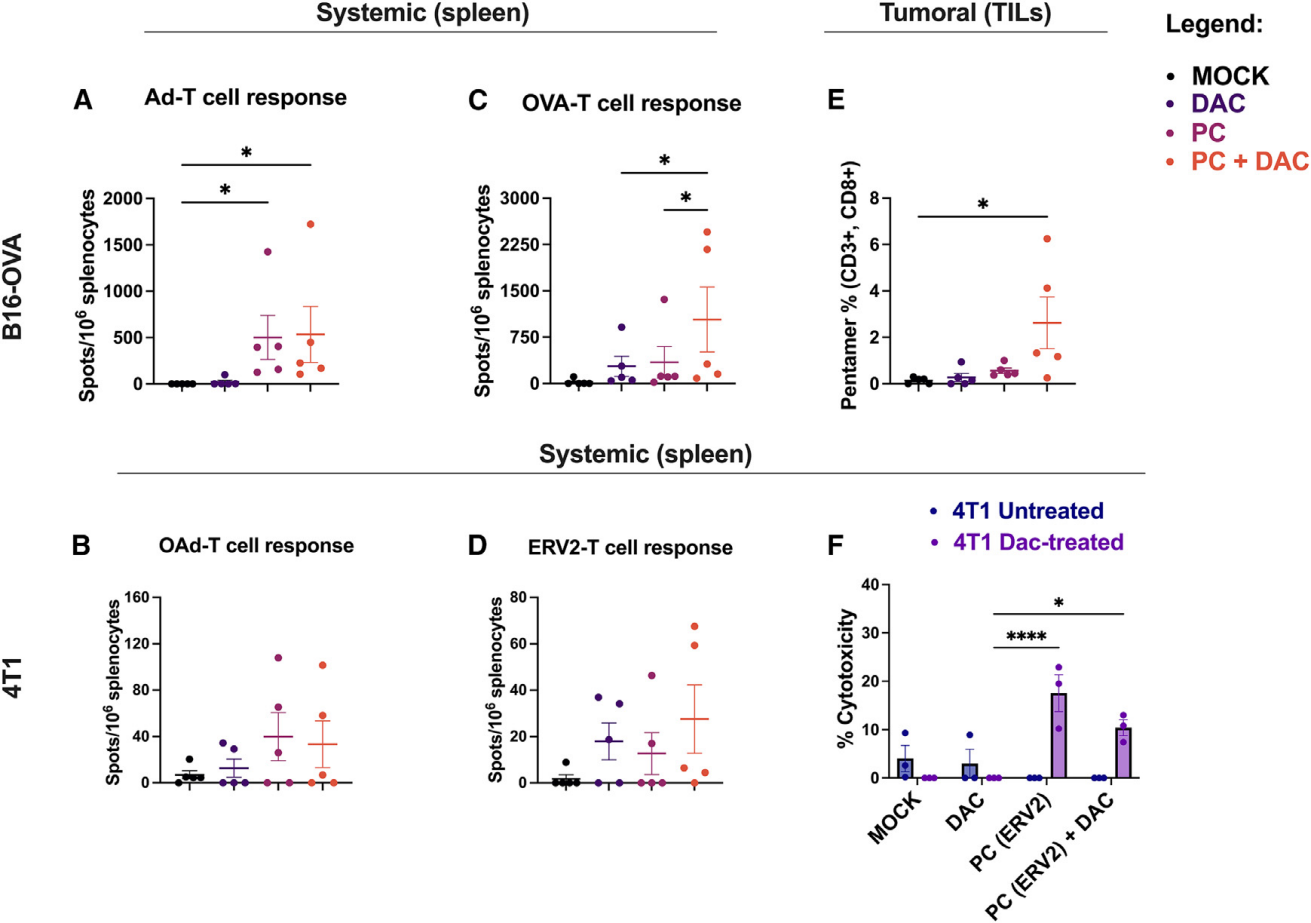
ELISpot IFN-γ analysis was performed on splenocytes harvested from B16-OVA or 4T1-bearing mice (A and B) In B16-OVA, splenocytes reactivity was tested against Ad-5/3 Δ24 (A) and OVA (SIINFEKL) (B). (C) The frequencies of T cell responses are depicted as spot-forming units per 1 × 10^6^ splenocytes; the amount of antitumor CD8^+^ TILs was measured by flow cytometry using APC-conjugated H-2Kb-SIINFEKL pentamer staining; each dot represents a single mouse; the mean is represented by the horizontal bar. (D and E) In 4T1, splenocytes reactivity was tested against Ad-5/3 Δ24 (D) and ERV2 (TYVAGDTQV) (E). Fresh splenocytes were harvested from five 4T1-bearing mice, and combined and cocultured with 4T1 cells previously treated with low-dose DAC. LDH release assay was performed on culture supernatant after 72 h. N = 5 in (A–E). N = 5 mice splenocytes were combined and analyzed in technical triplicates in (F). Levels of significance were set at ∗p < 0.05, ∗∗p < 0.01, ∗∗∗p < 0.001, and ∗∗∗∗p < 0.0001 (1-way ANOVA with Tukey’s multiple comparisons correction to compare individual groups). Graphs are shown as mean ± SEM.

Finally, we wanted to assess the efficacy of PeptiCRAd to elicit the peptide-specific T cell response in the context of an epigenetically remodeled TME. To this end, we analyzed systemic immunity (spleen) reactivity to OVA (SIINFEKL) and ERV2 (TYVAGDTQV) in the B16-OVA and 4T1 models, respectively, by IFN-γ enzyme-linked immunospot (ELISpot) assay. We additionally measured systemic reactivity to OAd as a control. As expected, we observed an increased antiadenoviral T cell response in the groups treated with PeptiCRAd as a single treatment or in combination with DAC in B16-OVA (Figure 6A), whereas only a modest but not significant response was recorded in 4T1 (Figure 6B) (Figure S7A). Similarly, an anti-OVA-specific T cell response was significantly induced in the B16-OVA mice receiving the combination (Figure 6C), and no differences were recorded between PC(ERV2) + DAC and the single agents in the 4T1 model (Figure 6D).

To link the effect of the treatments on the tumor growth and the immune profile of the mice, we checked the presence of specific SIINFEKL CD8^+^ T cells within B16-OVA tumors by pentamer H-2kb-SIINFEKL staining. Despite their being nonsignificant, we observed increased OVA-specific CD8^+^ T cell infiltration levels in PeptiCRAd (OVA)+ DAC-treated tumors (2.6%) rather than DAC alone (0.27%) and PeptiCRAd (OVA) alone (0.56%) (Figure 6E).

On the 4T1 side, to assess the cytotoxic capacity of antitumor T cells, we performed a killing assay by coculturing fresh murine splenocytes (effector cells) harvested from 4T1-bearing mice (MOCK, DAC, PeptiCRAd (ERV2), PeptiCRAd (ERV2)+ DAC) with DAC-treated or -untreated murine 4T1 cell line (target cells), and we measured lactate dehydrogenase (LDH) release after 72 h (Figure S7B). No relevant cytotoxicity was observed in untreated 4T1 cells in all of the groups (Figure 6F, blue bars) and effector cells alone in the presence or not of DAC (data not shown). However, we measured significant LDH release in the culture supernatant of PeptiCRAd (ERV2) and PeptiCRAd (ERV2)+ DAC murine splenocytes when incubated with DAC-treated 4T1 cells (17.5% and 10.4%, respectively) (Figure 6F, purple bars).

Overall, the data suggest that despite the presence of specific antitumor CD8^+^ T cells, as shown by the killing assay in the 4T1 model, the magnitude of the systemic antitumor response is cancer specific. Furthermore, as shown, antitumor T cells can induce direct anticancer cytotoxicity, however, upon epigenetic reprogramming of cancer cells.

## Discussion

In the present study, we evaluated whether the addition of epigenetic therapy could improve the efficacy of anticancer vaccines directed against murine melanoma and TNBC. We measured the expression of MHC class I and PD-L1 in cancer cell lines testing two different doses of DAC; 1 μM DAC is considered a clinically relevant dose in acute myeloid leukemia (AML) patients, linked to modest cell-type-dependent cytotoxicity and reduced impact on DNA methylation.^27^^,^^28^ However, 0.1 μM DAC is associated with improved immunological effects and no toxicity *in vitro*.^29^ As expected, we observed different sensitivities to DAC doses and IFN-γ according to the cell line species and the tumor type. In particular, this may be due to the different DNA methylation status and epigenetic regulations that cells have or acquire during the malignant progression.^30^ Confirming previous studies,^2^^,^^30^^,^^31^ here, we showed that DAC effectively induces MHC class I at the cell surface and further in the presence of IFN-γ in murine and human cancer cells. For this reason, from an immunological point of view, the combination of DAC or other epigenetic drugs with strategies that rely on the activation of cytotoxic lymphocytes (e.g., anticancer vaccines and adoptive T cell transfer) is extremely rational.

Therefore, we evaluated the low-dose epigenetic therapy and anticancer vaccines combined effect for improved response *in vivo*. It is important to consider that in this study, the effect of PeptiCRAd is independent of viral replication since the evaluation of murine models. However, PeptiCRAd preserves its anticancer vaccine activity to stimulate antitumor immune responses in mice. We observed that PeptiCRAd alone was able to reduce the intratumoral percentage of Tregs, as previously shown by studies performing intratumoral injections of OVs.^32^^,^^33^ Furthermore, the coadministration of PeptiCRAd and DAC promoted specific antitumor response in syngeneic B16-OVA without inducing a better control of the tumor growth over the single treatments. However, the same treatment induced higher tumor growth control and systemic immune activation in the 4T1 TNBC model. First, this effect may be justified by the increased MHC class I molecules on the tumor cells upon DAC treatment both *in vitro* and *in vivo*. Second, low-dose DAC induced a reduction in MDSCs in the 4T1 tumors, potentially decreasing the level of immunosuppression that heavily characterizes TNBCs. Previous studies have shown that depleting the MDSC population in the TME improve consistently the cancer sensitivity to cancer immunotherapy, representing an attractive strategy to increase the cohort of patients undergoing immunotherapy regimes. We also questioned whether DAC could intrinsically enhance the anticancer immune response induced by PeptiCRAd at a systemic level; however, the two models showed a different response magnitude as showed by the ELISpot assay. Again, we suggest that this phenomenon is linked to the different levels of immunosuppression that characterize the B16 melanoma and 4T1 TNBC. The local administration of PeptiCRAd in a highly immunosuppressive environment may have limited both the antitumor and antiviral immune responses in 4T1 compared to the more immunogenic B16-OVA. However, when cultured with DAC-primed 4T1 cells, splenocytes from PC(ERV2) alone and PC(ERV2) + DAC induced higher LDH release, suggesting that PeptiCRAd was able to increase antitumor response at a systemic level. In other words, DAC-mediated priming of 4T1 was strictly required to obtain a relevant anticancer response in the groups that have been treated with PC(ERV2); translated *in vivo*, this result correlates with reduced size of the 4T1 tumors in the PC(ERV2) + DAC group but not in ones receiving the single treatments. In addition, the combination group is associated with a general reduction in tumoral markers (pan-CK/E-cadherin) and, it is worth noting, heavy infiltration of CD8 cells in the pan-CK/E-cadherin^−^ areas. Overall, supported by the fact that the combination of DAC and OAd does not produce any relevant toxicity in murine cancer cells, these data suggest that the anticancer vaccine PeptiCRAd in combination with low-dose DAC exploits the tumor-associated immunity to promote tumor growth control. However, as shown in this study, this effect relies on the immunogenicity and sensitivity to DAC of the tumor.

In our model, however, we observed a suboptimal response. This phenomenon may rely on peptide selection because tumors show a heterogeneous repertoire of antigens through time and space. In this context, DAC is known to promote cancer testis antigen (CTA) expression, such as melanoma-associated antigen and New York esophageal squamous cell cancer-1 in cancer cells providing novel antigens/targets for cancer vaccines.^34^^,^^35^ In addition, as shown in this study and others, HMAs are associated with PD-L1 induction,^36^ potentially limiting the cytotoxic activity of CD8^+^ T cells against the tumor. In our 4T1 model, because of the increased PD-L1 levels upon DAC administration both *in vitro* and *in vivo* and the reduced coexpression of PD-1 and TIM-3 on CD8^+^ T cells, the addition of ICIs may further enhance the antitumor immune response provided by the combination of PeptiCRAd and DAC.^37^ Translated to clinical applications, evaluating cancer reactivity to epigenetic therapy and checkpoint inhibitors is crucial to developing more effective immunotherapies^13^^,^^14^ and expanding the eligibility of patients for inclusion in immunotherapy-based regimens.

Viral strategies have been previously tested in combination with epigenetic modulators. HMAs increase oncolytic herpes simplex virus (HSV) infection, replication, and persistence in brain and hematological malignancies *in vitro* and in mice models.^38^^,^^39^ However, most studies are focused on the effect of epigenetic drugs on viral-mediated oncolysis,^40^ whereas we explored the effect of low-dose epigenetic therapy on cancer immunogenicity in the context of cancer immunotherapy. To the best of our knowledge, only one study previously tested a similar approach, in which valproic acid, a histone deacetylase inhibitor, further improved a CD8^+^ T cell response against murine melanoma tumor-associated antigens (TAAs) initially primed by an HSV-expressing granulocyte macrophage-colony-stimulating factor.^41^ In our study, as an immunotherapy approach, we used PeptiCRAd, an OAd coated in TAs that is capable of eliciting potent antitumor responses. However, we could evaluate PeptiCRAd in humans since the oncolytic activity provided by the virus is chosen for a specific clinical application. For this reason, PeptiCRAd is a versatile platform that can be applied to other OVs in combination with a virtually limitless choice of tumor peptides. Nevertheless, although the choice of the OV may rely on differential cancer sensitivity, the identification and selection of reliable cancer antigens represent a bottleneck in human applications. The poor availability of human samples and the low mutational burden in some cancers considerably reduce the chances of finding relevant immunogenic tumor neoantigens. In these settings, TAAs are self-antigens that are aberrantly expressed in cancer cells and do not require patient-specific mutations to be recognized by the immune system and serve as attractive targets for immunotherapy. We and other groups have developed pipelines for the identification of TAAs from patient tumor samples,^42^^,^^43^^,^^44^ to increase the number of potential antigens to be used in clinical applications. Interestingly, HMAs have been shown to induce *de novo* expression of immunogenic CTAs and endogenous retroviral antigens in cancer cells.^45^^,^^46^ The selective induction of these antigens in cancer cells would increase the repertoire of targets to be recognized by CD8 T cells to mediate tumor rejection *in vivo* and reduce off-site effects that the involvement of TAAs would display. Despite being very attractive, more research is required to comprehensively assess the role of epigenetic and HMAs regulation of the antigen repertoire of cancer cells.^47^ In this context, despite not being well studied and characterized, it is important to mention that DAC (and probably other HMAs) can induce chromatin remodeling through DNA hypomethylation-independent mechanisms,^48^ affecting, therefore, the TME landscape in additional ways. The pleiotropic mode of action of DAC may account for the enhanced immune effects (e.g., neoantigen induction, increased immunogenicity) that were responsible in this and other studies for the improved cancer clearance or synergy with ICIs. Presently, however, the identification of the direct and indirect effects of HMAs on chromatin remodeling and the following gene expression is challenging and would require the use of models that are insensitive to DNA hypomethylation and the comparison among different HMAs agents in the context of cancer immunotherapy.

On the translational side, the evaluation of HMAs and OVs in humans would elucidate the feasibility and benefits of the strategy when applied to poorly immunogenic solid tumors. Both FDA-approved HMAs and FDA-approved talimogene laherparepvec (oncolytic herpesvirus)^49^ have been extensively studied in ICI-based treatments in solid tumors. However, no clinical trials are evaluating the two approaches in a single regimen. The recent FDA approval of the first adenoviral gene therapy for nonmuscle-invasive bladder cancer^50^ and the future development of viral therapies represent an opportunity to generate novel single or combinatorial approaches for cancer immunotherapy.

Finally, we observed differences between the intratumoral and spleen immune cell infiltration upon DAC systemic administration (Figures S4A and S4B). In the study performed by Wang et al., DAC effects were restricted mainly to the tumor tissue, rather than the spleen when injected systemically (i.p) in mice.^51^ In our study, although injected systemically at the low-dose level, the mode of action of DAC relies on DNA incorporation and blocking of DNA-methyltransferase activity. For this reason, replicating cells (as cancer cells) probably result as the main target of this drug. Changes in the whole cancer cells epigenome may also affect the composition of the TME and how the immune cells would interact with the cancer cells; this would explain why we observed different percentages in immune cell populations infiltrating the tumor and the spleen.

To conclude, our preclinical results support the use of HMAs in combination with viral vaccines for cancer immunotherapy. On one side, epigenetic therapy increases cancer immunogenicity and sensitivity to immunogenic cell death. On the other side, viral anticancer vaccines such as PeptiCRAd would provide the stimulation for building an antitumor immune response. Overall, our data show that the combination of PeptiCRAd + DAC is able to achieve this aim and correlates with the amelioration of tumor growth in syngeneic murine TNBC. However, as shown in the nonsynergistic effect in the B16 melanoma model, the success of the combination treatment relies on the tumor type and its reactivity to DAC.

## Materials and methods

### Cell lines

Human lung cancer A549 (American Type Culture Collection [ATCC] catalog no. CCL-185, Research Resource Identifier [RRID]: CVCL_0023), human TNBC MDA-MB-436 (ATCC catalog no. HTB-130, RRID: CVCL_0623), and human melanoma A-375 (ATCC catalog no. CRL-1619, RRID: CVCL_0132) cell lines were cultured in DMEM supplemented with 10% fetal bovine serum (FBS; Gibco), 1% glutamine (Gibco), 100 μg/mL streptomycin, and 100 U/mL penicillin (Life Technologies). Murine TNBC cell line 4T1 (ATCC catalog no. CRL-2539, RRID: CVCL_0125) was cultured in RPMI high glucose with 10% FBS (Gibco), 1% glutamine (Gibco), 100 μg/mL streptomycin, and 100 U/mL penicillin (Life Technologies). Prof. Richard Vile kindly provided B16-OVA, a mouse melanoma cell line expressing chicken OVA (Mayo Clinic). B16-OVA cells were cultured in RPMI with 10% FBS (Gibco), 1% glutamine (Gibco), 100 μg/mL streptomycin, and 100 U/mL penicillin (Life Technologies), and 5 mg/mL geneticin (Life Technologies). The cells were cultivated at 37°C, 5% CO_2_ in a humidified atmosphere, and regularly tested for mycoplasma contamination.

### OAds

The viral particle concentrations were measured by calculating the genome light absorbance at 260 nm. At the same time, the infection titer was determined by immunocytochemistry staining the adenoviral hexon protein on adenoviral-infected A549 cells. All of the adenoviruses were generated as conditionally replicating adenoviruses using standard protocols previously described.^15^

### Peptides

All of the peptides were purchased from Chempeptide. The following peptides were used for *ex vivo* splenocytes stimulation (in the ELISpot assay): SIINFEKL, SPSYAYHQ, FYLPTIRAV, and TYVAGDTQV. The following peptides were used for *in vivo* PeptiCRAd formulation: KKKKKKSIINFEKL, AH1: KKKKKKSPSYAYHQ, ERV1: KKKKKFYLPTIRAV, and ERV2: KKKKKKKTYVAGDTQV. More information regarding ERV1 and ERV2 identification and characterization can be found in Peltonen et al.^16^

### PeptiCRAd complex formation

The PeptiCRAd complex was prepared by mixing the OAd and each peptide with a polyK tail for 15 min at room temperature before intratumoral delivery. More details about PeptiCRAd can be found in Capasso et al.^15^

### Cell viability assay

A total of 10,000 cells were plated in a 96-well plate overnight and subsequently infected with 10-fold increasing MOI dilutions at different DAC concentrations. MTS (3-(4,5-dimethylthiazol-2-yl)-5-(3-carboxymethoxyphenyl)-2-(4-sulfophenyl)-2H-tetrazolium) determined cell viability according to the manufacturer’s protocol (Cell Titer 96 AQueous One Solution Cell Proliferation Assay, Promega, catalog no. G3580). Spectrophotometric data were acquired with Varioskan LUXMultimode Reader (Thermo Scientific) operated by SkanItsoftware.

### DAC experiments

Human or murine cancer cell lines were treated with 0.1 μM (low-dose) or 1 μM (high-dose) DAC (Selleck, catalog no. NSC 127716) for 4 days, followed by stimulation with human IFN-γ (100 ng/mL) (PeproTech, catalog no. 300-02) or murine IFN-γ (100 ng/mL) (Sigma Aldrich, catalog no. I4777-.1MG) Ad-5/3 Δ24 infection; at the end of the treatment, cells were analyzed by flow cytometry or by cell viability assay. Schematics of the DAC treatments are shown in Figures 1 and 2.

### DNA methylation quantification

DNA methylation of cancer cell lines was measured according to the Abcam Methylated DNA Quantification Kit.

### Animal experiments

All of the animal experiments were reviewed and approved by the Experimental Animal Committee of the University of Helsinki and the Provincial Government of Southern Finland (license no. ESAVI/11895/2019). The 4- to 6-week-old female Balb/cOlaHsd and C57BL/6 mice were obtained from Envigo Laboratory. The 3 × 10^5^ B16-OVA or 4T1 cells were injected s.c. into the right flank. Tumor-bearing mice were randomized before being assigned to a specific treatment group. The vaccine (viral dose was 1 × 10^9^ viral particles [vp]/tumor complexed with 20 μg of peptide) was given intratumorally (4 treatments), and 0.5 mg/kg DAC was given i.p. (5 treatments). Tumor growth was monitored every second day with a caliper and calculated using the following formula: (long side) × (short side)2/2. Animals were sacrificed when human ethical requirements were met. Tumors and spleens were harvested. Approximately 1-mm-long tissues were collected from the tumors and preserved in 10% formaldehyde for subsequent paraffin inclusion and IHC analysis.

### IFN-γ ELISpot

IFN-γ ELISpot assays were performed using commercially available mouse ELISpot reagent sets (ImmunoSpot), according to the manufacturer’s instructions. Spleens, collected at the endpoint of the experiment, were reduced to single-cell solution by passing them through a 70-μm strainer using the back of a syringe plunger. Following the manufacturer’s instructions, red blood cells were lysed using ACK buffer (Gibco, catalog no. A1049201). Splenocytes were then resuspended in Cellular Technology Limited (CTL) test medium (ImmunoSpot) and counted. For each well, 3 × 10^5^ splenocytes were seeded and were stimulated with 20 ng/μL (2 μg/well in total) of each peptide at 37°C for 72 h. After 3 days of stimulation, plates were developed, and the spot count was obtained using the CTL ImmunoSpot ELISpot plate reader system (ImmunoSpot).

### Flow cytometry

The anti-mouse reagents were TruStain FcX anti-mouse CD16/32 (BioLegend, catalog no. 156604, RRID: AB_2783138), APC-PD-L1 (BioLegend, catalog no. 374513, RRID: AB_2734441), FITC-H2Kb (BioLegend, catalog no. 116506, RRID: AB_313733), APC-H2Kd (BioLegend, catalog no. 116619, RRID: AB_10640118), PerCP/Cy5.5-EP-CAM (BioLegend, catalog no. 118219), PerCP/Cy5.5-CD44 (BioLegend, catalog no. 103032), FITC-CD11b (BioLegend, catalog no. 101206), PE-CD45 (BioLegend, catalog no. 103105), APC-Ly-6C (BioLegend, catalog no. 128015), BV510-Ly-6G (BD Horizon, catalog no. 74015), BV711-CD3 (BD Horizon, catalog no. 563123, RRID: AB_2687954), PE-CF594-CD4 (BD Horizon, catalog no. 562314, RRID: AB_11152957), FITC-CD8 (BioLegend, catalog no. 100706, RRID: AB_312745), PE-cy7-CD19 (BioLegend, catalog no. 302215, RRID: AB_314245), v450-CD25 (BD Horizon, catalog no. 561257, RRID: AB_10611871), PE-FOXP3 (BD Horizon, catalog no. 560408, RRID: AB_1645251), PE-PD-1 (BioLegend, catalog no. 135206, RRID: AB_1877231), PerCP/cy5.5-TIM-3 (BioLegend, catalog no. 119718, RRID: AB_2571935), and APC-CD3 (BioLegend, catalog no. 100235, RRID: AB_2561456). Apc-labeled H-2Kb/SIINFEKL pentamer + FITC-CD8 (ProImmune, catalog no. F093-84B-E) was used to measure the number of SIINFEKL epitope-specific T cells.

The anti-human reagents were PE-HLA-A, B, C (BioLegend, catalog no. 311406, RRID: AB_314875) and APC-PD-L1 (BioLegend, catalog no. 329708, RRID: AB_940360).

The data were acquired using BD LSRFortessa and ACCURI BD C6 plus flow cytometers and analyzed using FlowJo software version 9.

### Multiplexed fluorescence IHC (mfIHC)

Deparaffinized FFPE tissue sections (3.5 μm) mounted on microscopy slides (SuperFrost Ultra Plus/Fisher; J3800AMNZ) were processed with heat-induced epitope retrieval in 10 mM Tris-1 mM EDTA buffer (pH 9) for 20 min and then blocked for endogenous peroxidase in Tris-buffered saline (TBS) with 0.9% H_2_O_2_ for 15 min followed by protein blocking using TBS with 0.1% Tween 20 detergent with 10% normal goat serum. The mIHC primary antibodies were the following: CD8 (1:1,000, Cell Signaling Technology, catalog no. 98941, RRID: AB_2756376), CD31 (1:100, Abcam, catalog no. ab28364, RRID:AB_726362), pan-CK (1:100, Abcam, catalog no. ab9733), and E-cadherin (1:100, Cell Signaling Technology, catalog no. CST 3195). The secondary detection reagents were G-a-R-poly horseradish peroxidase (HRP) (Immunologic, catalog no. DPVR110HRP, RRID: AB_2915958) with Tyramide 488 (Life Technologies, catalog no. B40953) for CD8 detection. The CD31 and pan-CK/E-cadherin detection reagent was G-a-R-AF 750 (Life Technologies, catalog no. A21039, RRID: AB_2535710). Before pan-CK/E-cadherin antibody staining, slides were imaged and coverslips removed, followed by incubation in TBS/NaOH (24 mM)/H_2_O_2_ (4.5%) for 30–60 min (room temperature) and then in freshly prepared 10 mM Tris-1 mM EDTA pH 9 at 99°C for 20 min. Slides were counterstained with DAPI (1.6 μg/mL, Roche, catalog no. 10236276001) and mounted with ProLong Gold (Life Technologies, catalog no. P36934).

### Imaging

The whole-slide imaging was performed using a Zeiss Axio Scan.Z1 with Zeiss Plan-Apochromat objective 20× (0.8 NA, M27), Hamamatsu ORCA-Flash 4.0 V2 Digital CMOS camera (16-bit; 0.325 μm/pixel resolution), and Zeiss Colibri.7 LED Light Source. DAPI, fluorescein isothiocyanate, Cy5, and Cy7 filters were used.

### Image analysis

Following the whole-slide imaging, the multichannel fluorescence images were exported as single-channel grayscale images (64-bit, BigTiff format). Regions-of-interest with representative tumor areas excluding any artifacts (e.g., folded/ruptured tissue) were annotated and cropped using ImageJ (version 2.3.0 for Windows) with a Roi1 1-Click Tool plugin.

The final image analysis pipeline was performed using CellProfiler (version 4.2.1). Each marker was set as negative or positive by careful visual determination of the positivity threshold. The total number of marker-positive pixels was measured. Their relative numbers were counted by dividing them by the total number of nuclear DAPI pixels, generating results for relative cell areas.

### LDH assay

The killing was measured by determining the amount of LDH released using a colorimetric assay (CyQUANT LDH Cytotoxicity Assay, catalog no. C20303). The 4T1 cells were pretreated with 0.1 μM DAC as previously described in the paper. At the end of the treatment, a total of 10,000 untreated or DAC-treated 4T1 cells were seeded in a 96-well plate. Afterward, fresh Balb/c splenocytes (effector cells) (harvested from the 4T1 animal experiment) were added in a 100:1 ratio (effector:target ratio) and incubated for 72 h at 37°C. LDH was measured using the above-mentioned kit, and the percentage of cytotoxicity was calculated as follows: percentage cytotoxicity = (“experimental” − “effector + “target spontaneous”)/(“target maximum” – “target spontaneous”) × 100%, where “experimental” corresponds to the signal measured in a treated sample, “effector plus target spontaneous” corresponds to the signal measured in the presence of splenocytes and tumor cells alone individually, and “target maximum” corresponds to the signal measured in the presence of detergent-lysed tumor cells.

### Statistical analysis

Statistical analysis was performed using GraphPad Prism 9.0 software. Details about the statistical tests for each experiment can be found in the corresponding figure legends.

## Data and code availability

All of the data relevant to the study are included in the article or uploaded as supplementary information.

## Supplemental information

Document S1. Figures S1–S7

Document S2. Article plus supplemental information

## References

[1] Harrington K., Freeman D.J., Kelly B., Harper J., Soria J.-C. (2019). Optimizing oncolytic virotherapy in cancer treatment. Nat. Rev. Drug Discov..

[2] Taylor B.C., Balko J.M. (2022). Mechanisms of MHC-I Downregulation and Role in Immunotherapy Response. Front. Immunol..

[3] Cornel A.M., Mimpen I.L., Nierkens S. (2020). MHC Class I Downregulation in Cancer: Underlying Mechanisms and Potential Targets for Cancer Immunotherapy. Cancers.

[4] Li H., Chiappinelli K.B., Guzzetta A.A., Easwaran H., Yen R.-W.C., Vatapalli R., Topper M.J., Luo J., Connolly R.M., Azad N.S. (2014). Immune regulation by low doses of the DNA methyltransferase inhibitor 5-azacitidine in common human epithelial cancers. Oncotarget.

[5] Adair S.J., Hogan K.T. (2009). Treatment of ovarian cancer cell lines with 5-aza-2′-deoxycytidine upregulates the expression of cancer-testis antigens and class I major histocompatibility complex-encoded molecules. Cancer Immunol. Immunother..

[6] Pedroza-Gonzalez A., Kwekkeboom J., Sprengers D. (2013). T-cell suppression mediated by regulatory T cells infiltrating hepatic tumors can be overcome by GITRL treatment. OncoImmunology.

[7] Jones P.A., Issa J.-P.J., Baylin S. (2016). Targeting the cancer epigenome for therapy. Nat. Rev. Genet..

[8] Grunewald C.M., Schulz W.A., Skowron M.A., Hoffmann M.J., Niegisch G. (2018). Tumor immunotherapy—the potential of epigenetic drugs to overcome resistance. Transl. Cancer Res..

[9] Daver N., Boddu P., Garcia-Manero G., Yadav S.S., Sharma P., Allison J., Kantarjian H. (2018). Hypomethylating agents in combination with immune checkpoint inhibitors in acute myeloid leukemia and myelodysplastic syndromes. Leukemia.

[10] Wrangle J., Wang W., Koch A., Easwaran H., Mohammad H.P., Vendetti F., Vancriekinge W., Demeyer T., Du Z., Parsana P. (2013). Alterations of immune response of non-small cell lung cancer with Azacytidine. Oncotarget.

[11] Chiappinelli K.B., Strissel P.L., Desrichard A., Li H., Henke C., Akman B., Hein A., Rote N.S., Cope L.M., Snyder A. (2015). Inhibiting DNA Methylation Causes an Interferon Response in Cancer via dsRNA Including Endogenous Retroviruses. Cell.

[12] Terracina K.P., Graham L.J., Payne K.K., Manjili M.H., Baek A., Damle S.R., Bear H.D. (2016). DNA methyltransferase inhibition increases efficacy of adoptive cellular immunotherapy of murine breast cancer. Cancer Immunol. Immunother..

[13] Zeidan A.M., Boss I., Beach C.L., Copeland W.B., Thompson E., Fox B.A., Hasle V.E., Ogasawara K., Cavenagh J., Silverman L.R. (2022). A randomized phase 2 trial of azacitidine with or without durvalumab as first-line therapy for higher-risk myelodysplastic syndromes. Blood Adv..

[14] Goswami M., Gui G., Dillon L.W., Lindblad K.E., Thompson J., Valdez J., Kim D.-Y., Ghannam J.Y., Oetjen K.A., Destefano C.B. (2022). Pembrolizumab and decitabine for refractory or relapsed acute myeloid leukemia. J. Immunother. Cancer.

[15] Capasso C., Hirvinen M., Garofalo M., Romaniuk D., Kuryk L., Sarvela T., Vitale A., Antopolsky M., Magarkar A., Viitala T. (2016). Oncolytic adenoviruses coated with MHC-I tumor epitopes increase the antitumor immunity and efficacy against melanoma. OncoImmunology.

[16] Peltonen K., Feola S., Umer H.M., Chiaro J., Mermelekas G., Ylösmäki E., Pesonen S., Branca R.M.M., Lehtiö J., Cerullo V. (2021). Therapeutic Cancer Vaccination with Immunopeptidomics-Discovered Antigens Confers Protective Antitumor Efficacy. Cancers.

[17] Feola S., Russo S., Martins B., Lopes A., Vandermeulen G., Fluhler V., De Giorgi C., Fusciello M., Pesonen S., Ylösmäki E. (2022). Peptides-Coated Oncolytic Vaccines for Cancer Personalized Medicine. Front. Immunol..

[18] Ylösmäki E., Ylösmäki L., Fusciello M., Martins B., Ahokas P., Cojoc H., Uoti A., Feola S., Kreutzman A., Ranki T. (2021). Characterization of a novel OX40 ligand and CD40 ligand-expressing oncolytic adenovirus used in the PeptiCRAd cancer vaccine platform. Mol. Ther. Oncolytics.

[19] Hazini A., Fisher K., Seymour L. (2021). Deregulation of HLA-I in cancer and its central importance for immunotherapy. J. Immunother. Cancer.

[20] Garcia-Diaz A., Shin D.S., Moreno B.H., Saco J., Escuin-Ordinas H., Rodriguez G.A., Zaretsky J.M., Sun L., Hugo W., Wang X. (2017). Interferon Receptor Signaling Pathways Regulating PD-L1 and PD-L2 Expression. Cell Rep..

[21] Farrera-Sal M., Moya-Borrego L., Bazan-Peregrino M., Alemany R. (2021). Evolving Status of Clinical Immunotherapy with Oncolytic Adenovirus. Clin. Cancer Res..

[22] Feola S., Russo S., Ylösmäki E., Cerullo V. (2022). Oncolytic ImmunoViroTherapy: A long history of crosstalk between viruses and immune system for cancer treatment. Pharmacol. Ther..

[23] Lechner M.G., Karimi S.S., Barry-Holson K., Angell T.E., Murphy K.A., Church C.H., Ohlfest J.R., Hu P., Epstein A.L. (2013). Immunogenicity of Murine Solid Tumor Models as a Defining Feature of In Vivo Behavior and Response to Immunotherapy. J. Immunother..

[24] Jordan K.R., Mcmahan R.H., Kemmler C.B., Kappler J.W., Slansky J.E. (2010). Peptide vaccines prevent tumor growth by activating T cells that respond to native tumor antigens. Proc. Natl. Acad. Sci. USA.

[25] Loo Yau H., Bell E., Ettayebi I., De Almeida F.C., Boukhaled G.M., Shen S.Y., Allard D., Morancho B., Marhon S.A., Ishak C.A. (2021). DNA hypomethylating agents increase activation and cytolytic activity of CD8+ T cells. Mol. Cell.

[26] Lou Y., Preobrazhenska O., McDonald P.C., Roskelley C., Overall C.M., Dedhar S., auf dem Keller U., Sutcliffe M., McDonald P.C. (2008). Epithelial-mesenchymal transition (EMT) is not sufficient for spontaneous murine breast cancer metastasis. Dev. Dynam..

[27] Gu X., Tohme R., Tomlinson B., Sakre N., Hasipek M., Durkin L., Schuerger C., Grabowski D., Zidan A.M., Radivoyevitch T. (2021). Decitabine- and 5-azacytidine resistance emerges from adaptive responses of the pyrimidine metabolism network. Leukemia.

[28] Tsai H.-C., Li H., Van Neste L., Cai Y., Robert C., Rassool F.V., Shin J.J., Harbom K.M., Beaty R., Pappou E. (2012). Transient Low Doses of DNA-Demethylating Agents Exert Durable Antitumor Effects on Hematological and Epithelial Tumor Cells. Cancer Cell.

[29] Qin T., Youssef E.M., Jelinek J., Chen R., Yang A.S., Garcia-Manero G., Issa J.P.J. (2007). Effect of cytarabine and decitabine in combination in human leukemic cell lines. Clin. Cancer Res..

[30] Luo N., Nixon M.J., Gonzalez-Ericsson P.I., Sanchez V., Opalenik S.R., Li H., Zahnow C.A., Nickels M.L., Liu F., Tantawy M.N. (2018). DNA methyltransferase inhibition upregulates MHC-I to potentiate cytotoxic T lymphocyte responses in breast cancer. Nat. Commun..

[31] Stone M.L., Chiappinelli K.B., Li H., Murphy L.M., Travers M.E., Topper M.J., Mathios D., Lim M., Shih I.-M., Wang T.-L. (2017). Epigenetic therapy activates type I interferon signaling in murine ovarian cancer to reduce immunosuppression and tumor burden. Proc. Natl. Acad. Sci. USA.

[32] Feist M., Zhu Z., Dai E., Ma C., Liu Z., Giehl E., Ravindranathan R., Kowalsky S.J., Obermajer N., Kammula U.S. (2021). Oncolytic virus promotes tumor-reactive infiltrating lymphocytes for adoptive cell therapy. Cancer Gene Ther..

[33] Kaufman H.L., Kim D.W., Deraffele G., Mitcham J., Coffin R.S., Kim-Schulze S. (2010). Local and Distant Immunity Induced by Intralesional Vaccination with an Oncolytic Herpes Virus Encoding GM-CSF in Patients with Stage IIIc and IV Melanoma. Ann. Surg Oncol..

[34] Yu G., Wang W., He X., Xu J., Xu R., Wan T., Wu Y. (2022). Synergistic Therapeutic Effects of Low Dose Decitabine and NY-ESO-1 Specific TCR-T Cells for the Colorectal Cancer With Microsatellite Stability. Front. Oncol..

[35] Bi S.Q., Zhang Q.M., Zeng X., Liu C., Nong W.X., Xie H., Li F., Lin L.N., Luo B., Ge Y.Y., Xie X.X. (2022). Combined treatment with epigenetic agents enhances anti-tumor activity of MAGE-D4 peptide-specific T cells by upregulating the MAGE-D4 expression in glioma. Front. Oncol..

[36] Pardoll D.M. (2012). The blockade of immune checkpoints in cancer immunotherapy. Nat. Rev. Cancer.

[37] Wu S.-Y., Xiao Y., Wei J.-L., Xu X.-E., Jin X., Hu X., Li D.-Q., Jiang Y.-Z., Shao Z.-M. (2021). MYC suppresses STING-dependent innate immunity by transcriptionally upregulating DNMT1 in triple-negative breast cancer. J. Immunother. Cancer.

[38] Ishino R., Kawase Y., Kitawaki T., Sugimoto N., Oku M., Uchida S., Imataki O., Matsuoka A., Taoka T., Kawakami K. (2021). Oncolytic Virus Therapy with HSV-1 for Hematological Malignancies. Mol. Ther..

[39] Okemoto K., Kasai K., Wagner B., Haseley A., Meisen H., Bolyard C., Mo X., Wehr A., Lehman A., Fernandez S. (2013). DNA demethylating agents synergize with oncolytic HSV1 against malignant gliomas. Clin. Cancer Res..

[40] Forbes N.E., Abdelbary H., Lupien M., Bell J.C., Diallo J.S. (2013). Exploiting tumor epigenetics to improve oncolytic virotherapy. Front. Genet..

[41] Jennings V.A., Scott G.B., Rose A.M.S., Scott K.J., Migneco G., Keller B., Reilly K., Donnelly O., Peach H., Dewar D. (2019). Potentiating Oncolytic Virus-Induced Immune-Mediated Tumor Cell Killing Using Histone Deacetylase Inhibition. Mol. Ther..

[42] Feola S., Chiaro J., Martins B., Russo S., Fusciello M., Ylösmäki E., Bonini C., Ruggiero E., Hamdan F., Feodoroff M. (2022). A novel immunopeptidomic-based pipeline for the generation of personalized oncolytic cancer vaccines. Elife.

[43] Chiaro J., Antignani G., Feola S., Feodoroff M., Martins B., Cojoc H., Russo S., Fusciello M., Hamdan F., Ferrari V. (2023). Development of mesothelioma-specific oncolytic immunotherapy enabled by immunopeptidomics of murine and human mesothelioma tumors. Nat. Commun..

[44] Zhang B., Bassani-Sternberg M. (2023). Current perspectives on mass spectrometry-based immunopeptidomics: the computational angle to tumor antigen discovery. J. Immunother. Cancer.

[45] Guo Z.S., Hong J.A., Irvine K.R., Chen G.A., Spiess P.J., Liu Y., Zeng G., Wunderlich J.R., Nguyen D.M., Restifo N.P., Schrump D.S. (2006). De novo induction of a cancer/testis antigen by 5-aza-2'-deoxycytidine augments adoptive immunotherapy in a murine tumor model. Cancer Res..

[46] Ku Y., Park J.-H., Cho R., Lee Y., Park H.-M., Kim M., Hur K., Byun S.Y., Liu J., Lee Y.-S. (2021). Noncanonical immune response to the inhibition of DNA methylation by Staufen1 via stabilization of endogenous retrovirus RNAs. Proc. Natl. Acad. Sci. USA.

[47] Chong C., Coukos G., Bassani-Sternberg M. (2022). Identification of tumor antigens with immunopeptidomics. Nat. Biotechnol..

[48] Jabbour E., Issa J.P., Garcia-Manero G., Kantarjian H. (2008). Evolution of decitabine development. Cancer.

[49] Ferrucci P.F., Pala L., Conforti F., Cocorocchio E. (2021). Talimogene Laherparepvec (T-VEC): An Intralesional Cancer Immunotherapy for Advanced Melanoma. Cancers.

[50] (2022). FDA Approves First Adenoviral Vector-Based Gene Therapy for High-Risk Bacillus Calmette-Guérin Unresponsive Non-muscle Invasive Bladder Cancer. https://www.fda.gov/drugs/resources-information-approved-drugs/fda-approves-first-adenoviral-vector-based-gene-therapy-high-risk-bacillus-calmette-guerin.

[51] Wang L., Amoozgar Z., Huang J., Saleh M.H., Xing D., Orsulic S., Goldberg M.S. (2015). Decitabine Enhances Lymphocyte Migration and Function and Synergizes with CTLA-4 Blockade in a Murine Ovarian Cancer Model. Cancer Immunol. Res..

