# Supplementary material for: Low-dose decitabine enhances the efficacy of viral cancer vaccines for immunotherapy
Source: Mol Ther Oncol. 2024 Jan 26;32(1):200766. doi: 10.1016/j.omton.2024.200766 (PMC10869747; doi:10.1016/j.omton.2024.200766)
Supplement: Document S1. Figures S1–S7 [file mmc1.pdf]

OMTON, Volume *32*

Supplemental information

Low-dose decitabine enhances the efficacy of viral cancer vaccines for immunotherapy

Salvatore Russo, Sara Feola, Michaela Feodoroff, Jacopo Chiaro, Gabriella Antignani, Manlio Fusciello, Federica D'Alessio, Firas Hamdan, Teijo Pellinen, Riikka Mölsä, Lorella Tripodi, Lucio Pastore, Mikaela Grönholm, and Vincenzo Cerullo

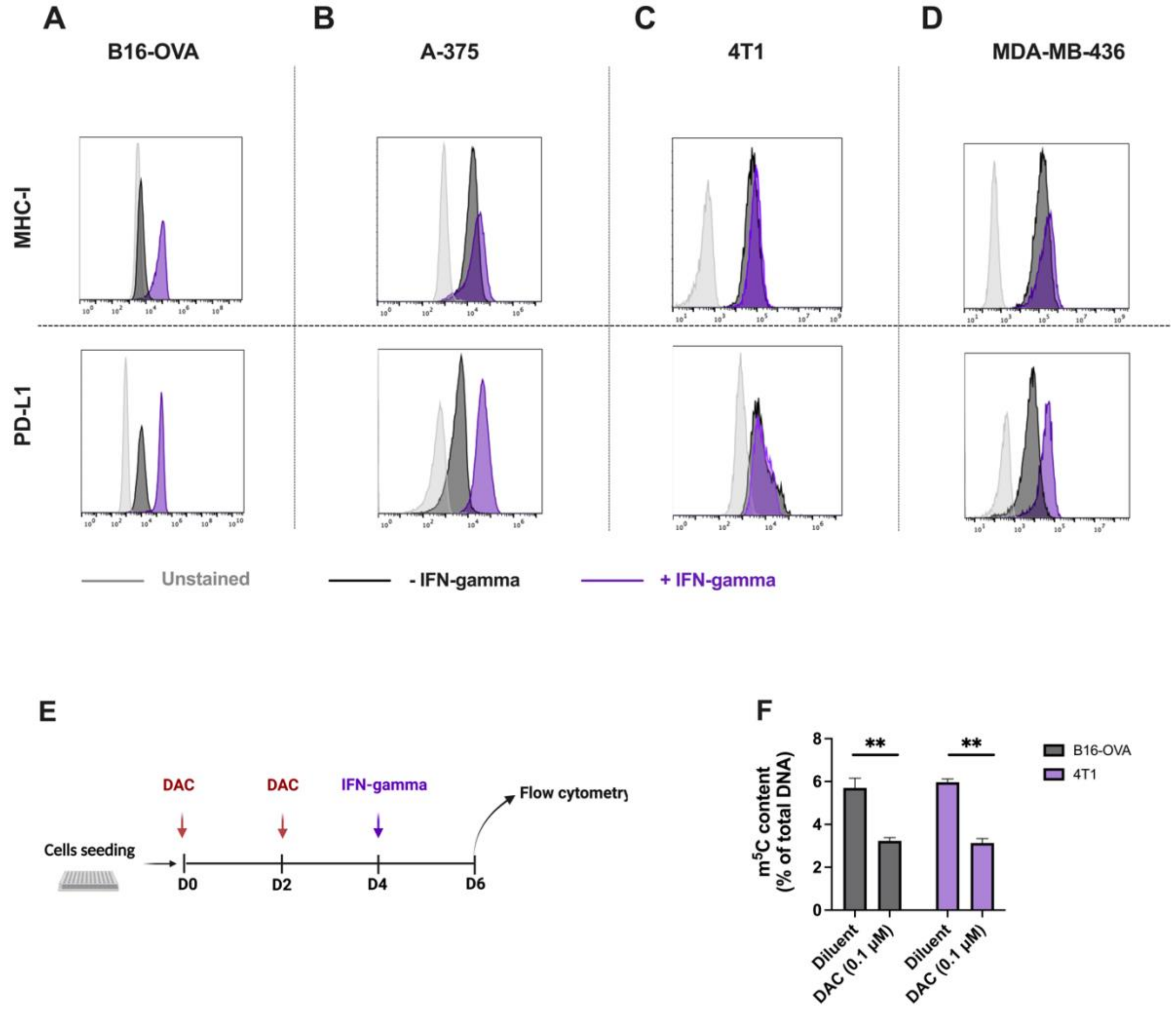

Figure S1

Histogram plot representing MHC-I or PD-L1 fluorescence intensity lines after IFN-gamma stimulation. *B16-OVA (**A**), A-375 (**B**), 4T1 **(C),** and MDA-MB-436 (**D**) cell lines were analyzed by flow cytometry using anti-mouse or anti-human fluorophore-conjugated antibodies against MHC-I or PD-L1. **E**) Treatment schedule of cancer cell lines. **F**) Global m5C content in DNA from B16-OVA and 4T1 cell lines was examined by an ELISA assay. Levels of significance were set at *p<0.05, **p<0.01, ***p<0.001, and ****p<0.0001 (two-way ANOVA with Tukey's multiple comparisons correction to compare individual groups). Graphs are shown as mean ± SD.*

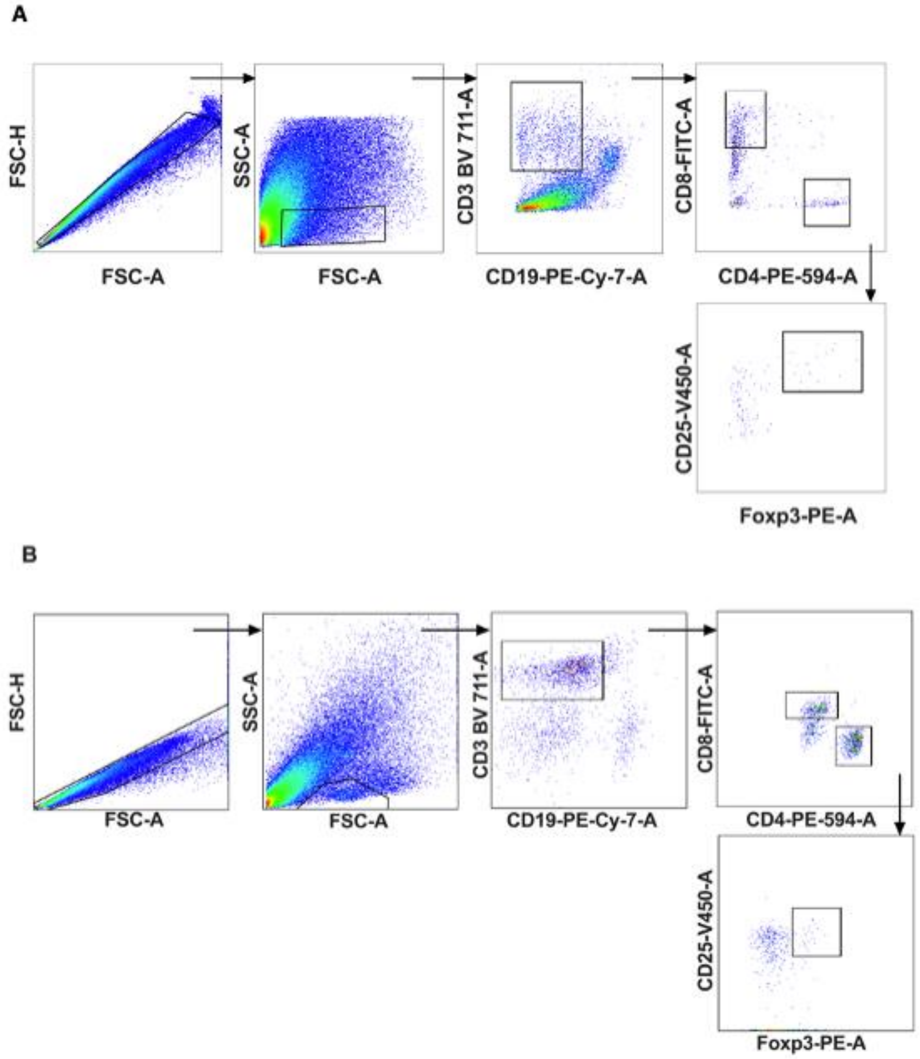

Figure S2

Depiction of the gating strategy for flow cytometry of tumors.

*Representative flow cytometry plots for CD4+, CD8+ T cells, and Tregs (Foxp3+ and CD25+) in B16-OVA (**A**) and 4T1 (**B**).*

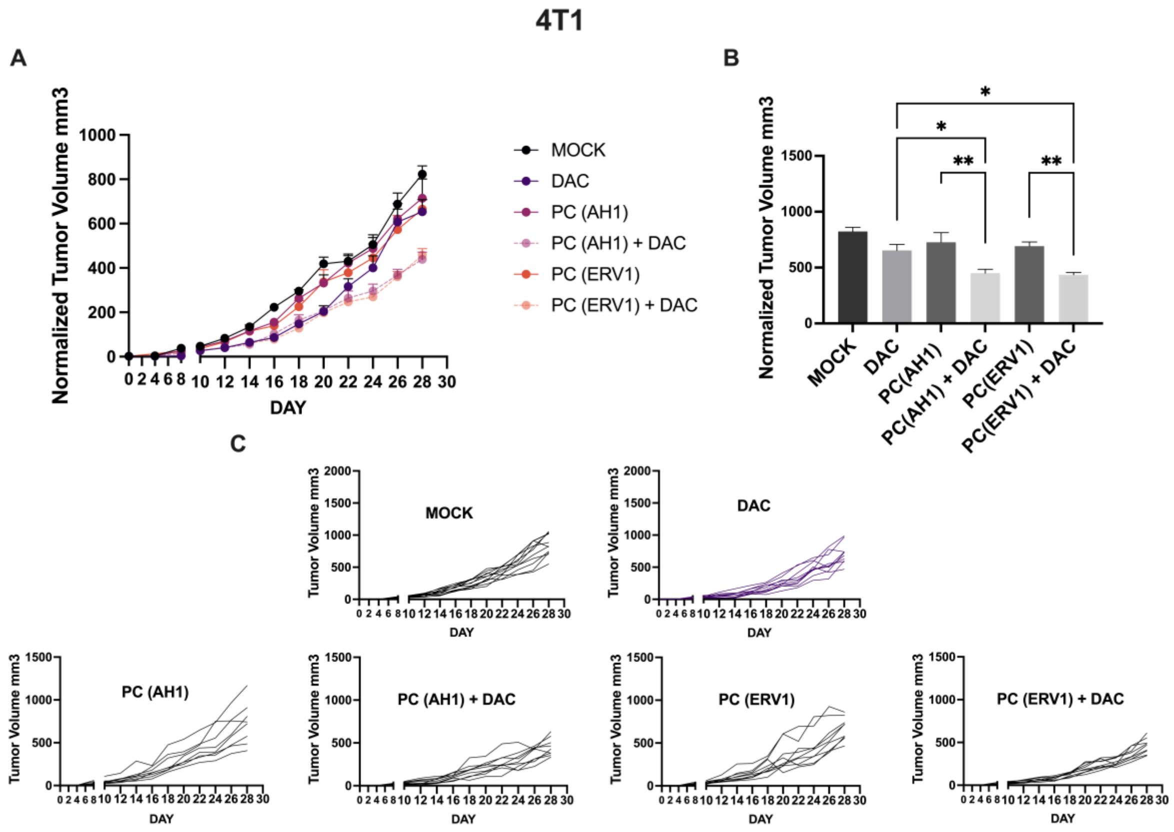

Figure S3

Tumor growth in a syngeneic mouse model of 4T1.

Normalized tumor growth curve and volumes reached at day 28 are shown in ***A*** *and* ***B*** *respectively***.** *Tumor volumes were normalized on day 4 measurements. The picture in* ***C*** *contains single tumor growth curves for each treatment group; the number of mice in the 4T1 model was 8-10. Levels of significance were set at *p<0.05, **p<0.01, ***p<0.001, and ****p<0.0001 (two-way ANOVA with Tukey's multiple comparisons correction to compare individual groups). Bars represent SEM.*

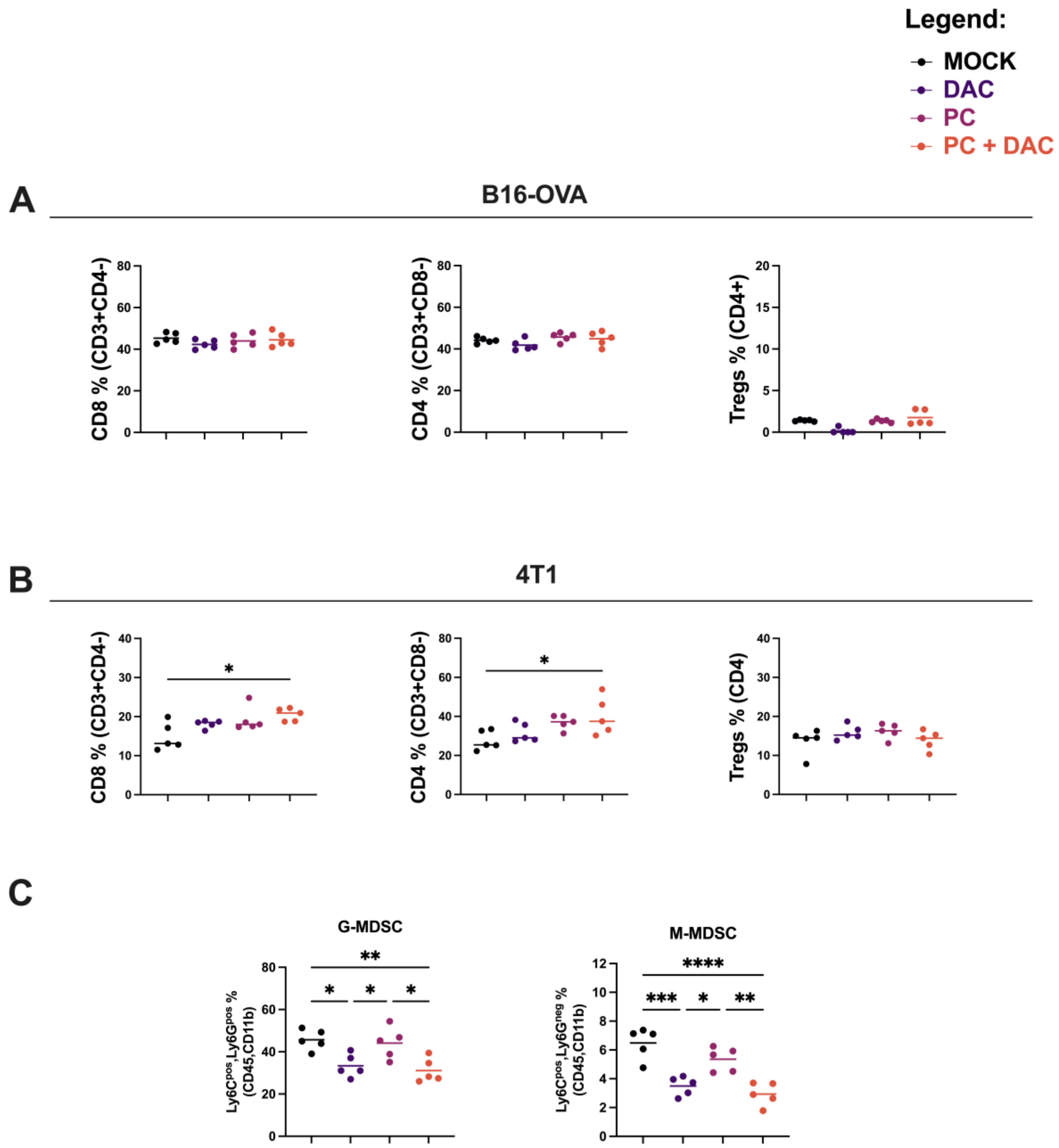

Figure S4

Immunological analysis of the spleen in syngeneic mouse model B16-OVA melanoma and 4T1 TNBC.

*CD8, CD4, and Tregs (Foxp3 and CD25 positive) markers were evaluated by flow cytometry in the spleen (one dot = one mouse, biological replicate) from B16-OVA (**A**) and 4T1 (**B**). Intratumoral percentage of granulocytic MDSCs (G-MDSCs) and monocytic MDSCs (M-MDSCs) in 4T1 tumors. Levels of significance were set at *p<0.05, **p<0.01, ***p<0.001, and ****p<0.0001 (one-way ANOVA with Tukey's multiple comparisons correction to compare individual groups). Bars represent the mean.*

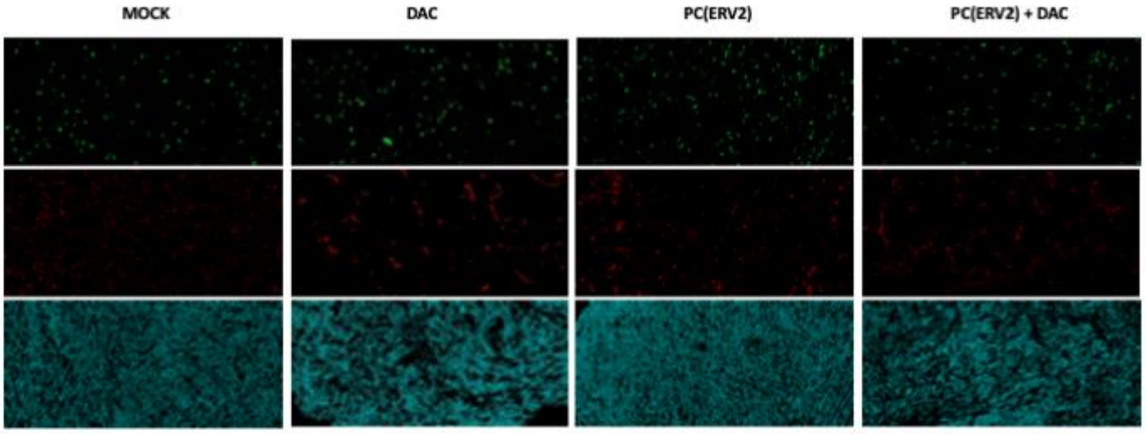

Figure S5

Representative images from high-density panCK/E-cadherin areas within the 4T1 murine tumors. Tumor slides were antibody-stained with lymphocyte (CD8), endothelial (CD31), and tumor (pan-cytokeratin and E-cadherin) markers.

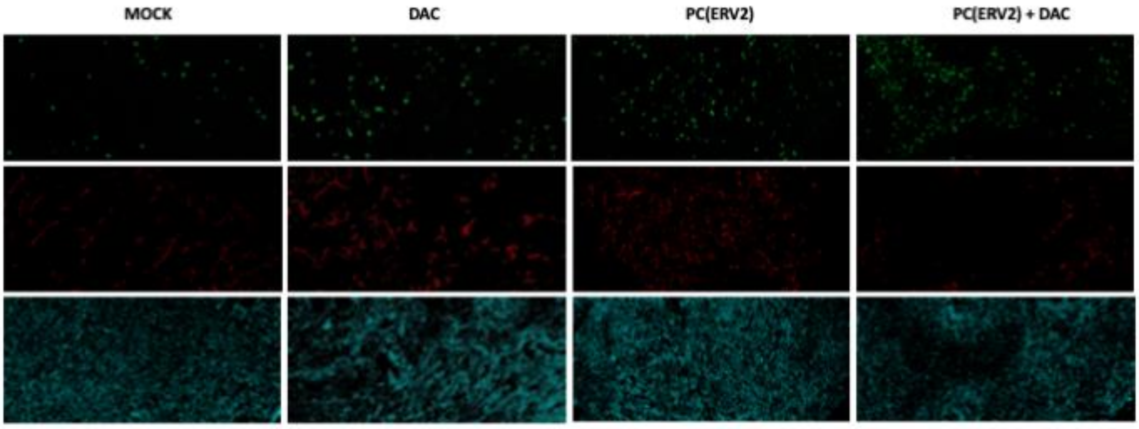

Figure S6

Representative images from low-density panCK/E-cadherin areas within the 4T1 murine tumors. Tumor slides were antibody-stained with lymphocyte (CD8), endothelial (CD31), and tumor (pan-cytokeratin and E-cadherin) markers.

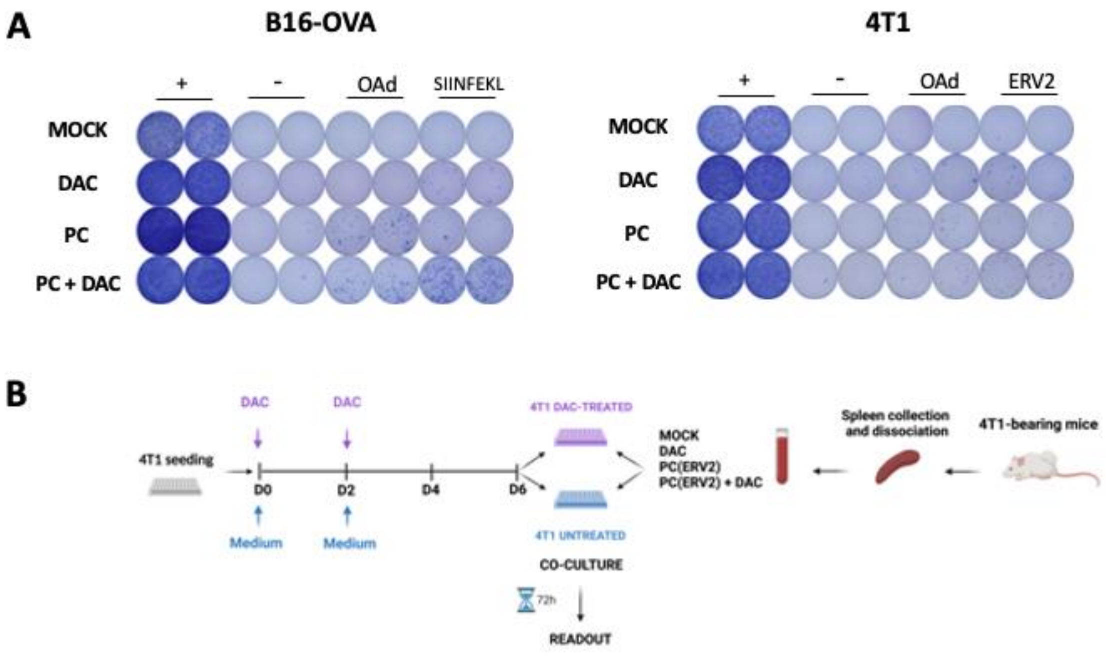

Figure S7

ELISPOT representative readouts are shown in ***A.*** *Experimental design of the killing assay is depicted in* ***B.***
